# Design principles to assemble drug combinations for effective tuberculosis therapy using interpretable pairwise drug response measurements

**DOI:** 10.1016/j.xcrm.2022.100737

**Published:** 2022-09-08

**Authors:** Jonah Larkins-Ford, Yonatan N. Degefu, Nhi Van, Artem Sokolov, Bree B. Aldridge

**Affiliations:** 1 Department of Molecular Biology and Microbiology, Tufts University School of Medicine, Boston, MA 02111, USA; 2 Stuart B. Levy Center for Integrated Management of Antimicrobial Resistance, Boston, MA 02111, USA; 3 Graduate School of Biomedical Sciences, Tufts University School of Medicine, Boston, MA 02111, USA; 4 Laboratory of Systems Pharmacology, Harvard Program in Therapeutic Science, Harvard Medical School, Boston, MA 02115, USA; 5 Department of Biomedical Engineering, Tufts University School of Engineering, Medford, MA 02155, USA

**Keywords:** tuberculosis, microbiology, combination therapy, machine learning, multidrug therapy, antibiotics, infectious diseases, drug interactions

## Abstract

A challenge in tuberculosis treatment regimen design is the necessity to combine three or more antibiotics. We narrow the prohibitively large search space by breaking down high-order drug combinations into drug pair units. Using pairwise *in vitro* measurements, we train machine learning models to predict higher-order combination treatment outcomes in the relapsing BALB/c mouse model. Classifiers perform well and predict many of the >500 possible combinations among 12 antibiotics to be improved over bedaquiline + pretomanid + linezolid, a treatment-shortening regimen compared with the standard of care in mice. We reformulate classifiers as simple rulesets to reveal guiding principles of constructing combination therapies for both preclinical and clinical outcomes. One example ruleset combines a drug pair that is synergistic in a dormancy model with a pair that is potent in a cholesterol-rich growth environment. These rulesets are predictive, intuitive, and practical, thus enabling rational construction of drug combinations.

## Highlights

• Evaluate the large drug combination space for potential tuberculosis treatments
• *In vitro* 2-drug combination measurements predict 3–4 drug treatment outcomes *in vivo*
• Strongly synergistic, antagonistic, or potent drug pairs drive treatment outcome
• Simple rules articulate drug combination design principles for tuberculosis

## Graphical abstract

**Figure:**
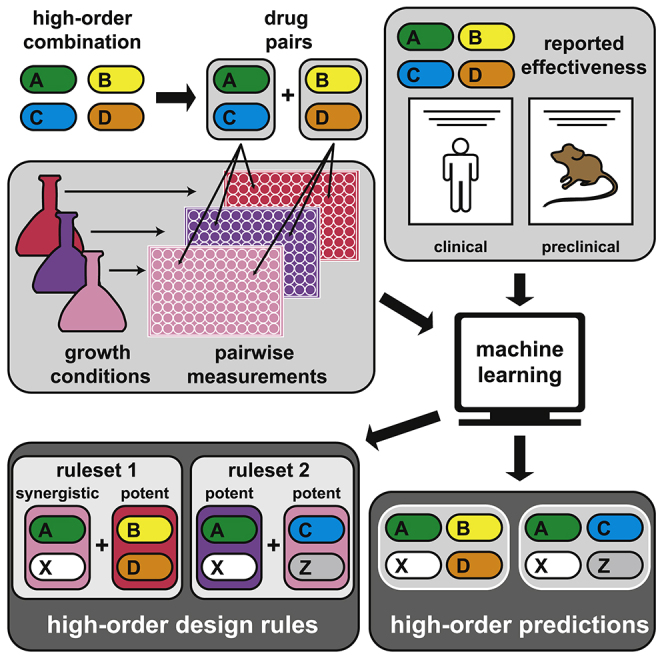

## Teaser

Larkins-Ford et al. describe an efficient and interpretable method to evaluate drug combinations early in drug regimen design for tuberculosis. Using units of pairwise drug combination measurements *in vitro*, they predict multidrug treatment outcomes *in vivo* and describe what properties of pairwise building blocks are required for effective multidrug therapies.

## Introduction

Tuberculosis (TB) remains an important global health concern, with more than 10 million people falling ill and about 1.4 million dying in 2019.^1^ Multiple drugs are used to treat TB because combination therapy shortens treatment duration, reduces disease relapse, and lowers the rate of drug resistance development compared with monotherapy.^2^ The standard of care (SOC) for treatment was developed almost 40 years ago and consists of four drugs (isoniazid [H], rifampicin [R], pyrazinamide [Z], ethambutol [E] [HRZE]) given for 2 months followed by two drugs (H and R; HR) given for another 4–7 months.^2^ New multidrug therapies are needed to improve outcomes and should include drugs that shorten treatment, increase efficacy, or both.

Efforts to develop new antibiotics and combination therapies for TB have been highly productive (https://www.newtbdrugs.org),^3^ but the large combination space cannot be surveyed clinically. Improved drug combinations may be in this space, as a recent clinical study identified a four-drug combination that shortened treatment by 2 months by substituting two drugs (H and R) from the SOC with moxifloxacin (M) and rifapentine (P).^4^ Furthermore, the combination consisting of bedaquiline (B), pretomanid (Pa), and linezolid (L) (BPaL) has become an example for attainable treatment improvement because it shortened treatment of multidrug-resistant TB (MDR-TB) from over 2 years to 6 months with increased efficacy from <50% to >90% cure.^5^ Reciprocal methods to clinical studies are needed to design combination therapies rapidly and systematically.

Preclinical animal studies are primary tools to identify drug combinations for clinical evaluation. The BALB/c relapsing mouse model (RMM) identified BPaL as a highly effective combination that showed faster and more effective cure for treating drug-sensitive *Mycobacterium tuberculosis* than the three-drug mouse SOC (HRZ),^6^ highlighting the utility of the RMM to identify treatment-shortening combinations for drug-sensitive or drug-resistant *M. tuberculosis*. However, the number of combinations that can be tested in mouse studies is limited, and methods that prioritize drug combinations for preclinical testing are needed. We recently demonstrated that *in vitro* drug measurements in suites of multiple growth conditions were predictive of treatment improvement over the SOC in the RMM,^7^ suggesting a path forward to prioritize drug combinations.

**Figure 1:**
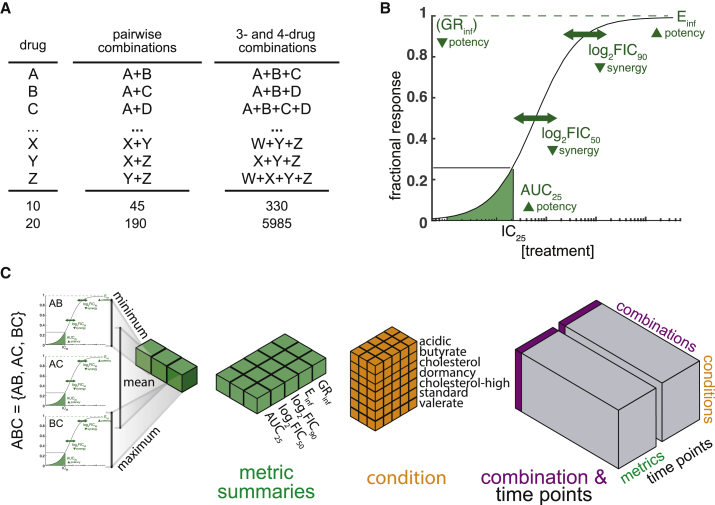
Data structure to organize *in vitro* drug-pair data underlying higher-order drug combinations (A) Summary of combinatorial explosion going from single drugs to three- and four-drug combinations for 10 and 20 drugs. (B) Diagram of drug combination dose-response curve, highlighting four (E_inf_, log_2_FIC_90_, log_2_FIC_50_, AUC_25_) of the five metrics calculated. GR_inf_ is not diagramed because a separate dose-response curve is used.^12^ Below each metric is an arrow that points to whether low (down arrow) or high (up arrow) metric values are potent or synergistic. (C) Diagram of data structure used in the study. Combination ABC is composed of three drug pairs: AB, AC, and BC. Metrics from each pairwise dose-response curve are collated and summarized by calculating the minimum, maximum, and mean for each metric (green) for every measured growth condition. The summary metrics for a combination in an *in vitro* condition (orange) are compiled and concatenated with the metrics for all *in vitro* conditions (purple) to constitute all the pairwise data underlying a high-order combination. The totality of data from all combinations (gray) at two time points in seven growth conditions and five metrics comprise this *in vitro* dataset.

One approach to efficiently search the drug combination space is to utilize drug-pair data instead of empirical measurement of three- and four-way combinations (Figure 1A). For example, there are almost 6,000 three- and four-drug combinations among 20 drugs but only 190 drug pairs; therefore, a method based on pairwise measurements would improve efficiency by ∼30-fold. The *in vitro* behavior of high-order drug combinations (three or more drugs) can be predicted from the underlying low-order combinations,^8^, ^9^, ^10^, ^11^ indicating that information important for understanding high-order activity is contained in drug-pair measurements. These methods were developed to investigate drug interactions, which describe how drugs in combination interact to produce effects that are greater than, less than, or as good as the effects of individual drugs (synergy, antagonism, and additivity, respectively). The success in mapping pairwise to high-order drug interactions *in vitro* suggests the possibility to predict outcomes of multidrug therapies *in vivo* based on the properties of underlying drug pairs.

Our goal is to fill a gap in the drug combination development pipeline by evaluating the vast number of candidate combinations early in drug regimen design. We aim to prioritize combinations for resource-intensive *in vivo* studies and dose optimization (Figure S1). Our study design is motivated by (1) the predictive signal of *in vitro* drug combination measurements in the RMM, and (2) the ability to predict high-order drug interactions from underlying pairwise interactions. Using systematic pairwise drug response data, we developed machine learning (ML) models that accurately predict RMM and clinical outcomes of high-order combinations, creating a scalable and resource-sparing method to design combination therapies. We found that pairwise *in vitro* data carry a strong predictive signal, and that building blocks of drug pairs form the basis of interpretable rulesets for constructing effective high-order combinations. Furthermore, the combination design principles translated from the RMM to clinical outcomes. Our framework simultaneously creates accurate predictions of combination therapy outcomes in preclinical models and interpretable rules to construct optimized combinations.

## Results

### Organizing high-order drug combinations by summarizing pairwise drug combination data

We hypothesized that we could predict high-order drug combination RMM treatment outcomes using *in vitro* pairwise drug combination measurements because pairwise drug interaction data are predictive of high-order drug interactions in *M. tuberculosis*, *Escherichia coli*, and cancer cells.^8^, ^9^, ^10^^,^^13^ In addition, we previously showed that *in vitro* drug combination response data are predictive of RMM treatment outcomes.^7^ To test this hypothesis, we designed a data structure to organize pairwise *in vitro* drug combination measurements across a range of drug-pair potencies and drug interactions for each high-order drug combination under consideration (Figure 1).

We used pairwise drug combination response data from a large-scale study that contains *in vitro* measurement of two- and three-drug combinations among 10 commonly used anti-TB drugs.^7^ We expanded this 10-drug set (B, clofazimine, E, H, L, M, Pa, Z, P, R) with pairwise measurement to include SQ109 and sutezolid, for a total of 12 drugs (Table 1). A portion of the SQ109 pairwise data was described previously,^14^ while its remainder and all the sutezolid data are new to this study. An equipotent mixture of each drug was measured at multiple doses to generate a pairwise dose-response curve.^7^^,^^11^^,^^15^ Drug combinations were measured in seven *in vitro* growth conditions relevant to the environments encountered by *M. tuberculosis* during infection (Table 1): fatty acid carbon sources consisting of (1) butyrate, (2) valerate, (3) 0.05 mM cholesterol, and (4) 0.2 mM cholesterol (cholesterol-high), as well as (5) acidic medium (acidic), (6) non-replicating/hypoxic medium (dormancy), and (7) standard laboratory growth medium (standard). *M. tuberculosis* replicate during incubation in all conditions except dormancy, which induces a metabolically inactive, non-replicative state.^7^^,^^14^ Longitudinal measurements were made, and two time points were targeted that represent a relatively consistent drug exposure time across conditions (constant), as well as the maximal drug exposure time relative to the doubling time of *M. tuberculosis* in each growth condition (terminal; constant and terminal times were the same for the standard condition, Table 1). Five metrics were calculated for each dose-response curve (Figure 1B and Table 1), capturing combination potency (the normalized area under the dose-response curve up to the 25% growth inhibitory concentration [IC_25_] [AUC_25_], effect at infinite drug concentration [maximum achievable effect] [E_inf_], normalized growth inhibition effect at infinite drug concentration [maximum achievable effect] [GR_inf_]) and drug interactions at low and high doses (fractional inhibitory concentration at 50% growth inhibition [log_2_FIC_50_], fractional inhibitory concentration at 90% growth inhibition [log_2_FIC_90_]). In total, 65 metrics were calculated for each of the 60 drug pairs, totaling 3,900 pairwise dose-response metrics (Data S1).

When breaking down high-order drug combinations into corresponding drug pair sets (e.g., ABC into AB, AC, and BC), some drug pairs will serve as components of multiple high-order drug combinations (e.g., AB is a component of ABC, ABD, ABCD). An important consequence is that each drug pair in a high-order drug combination will have an associated metric (e.g., for combination ABC, there will be an E_inf_ metric for AB, AC, and BC), but drug combinations of different orders will consist of different numbers of drug pairs and consequently have different numbers of pairwise dose-response metrics. To make combinations of different orders comparable, we devised a data structure where each high-order drug combination was represented by the same number of dose-response features, accomplished by aggregating the constituent pairwise metrics (AUC_25_, E_inf_, GR_inf_, log_2_FIC_50_, log_2_FIC_90_) using three summary statistics: minimum (min), maximum (max), and arithmetic mean (mean; Figure 1C). The three summary statistics ensured a uniform data structure of 195 features (mean, min, max of pairs for each metric, condition, and time point) from pairwise data for all high-order combinations (Data S1), facilitating downstream analyses.

### Pairwise data are predictive of high-order in vivo treatment outcome

To test the hypothesis that *in vivo* high-order drug combination treatment outcomes can be predicted from *in vitro* pairwise treatment data, we binned the dataset of high-order (three-, four-, and five-drug) combinations by assessing whether each combination was better (+C1) or not (−C1) than the SOC in the RMM outcome using published animal studies. In brief, combinations were deemed better than the SOC if they achieved lower relapse (increased efficacy), similar relapse percentage with shorter treatment time (treatment shortening), or both (Data S2; see STAR Methods for details on annotation and binning). These binned annotations were consistent with the combination treatment improvement estimated in an interstudy comparison using a mixed-effects logistic regression model approach to normalize the differences in study methodologies.^16^ Principal-component (PC) analysis (PCA) revealed partial separation of +C1 and −C1 combinations along the first PC, indicating a strong predictive signal in pairwise data and suggesting that linear combinations of *in vitro* pairwise drug responses may be sufficient to distinguish drug combinations with different *in vivo* outcomes, even in the absence of trained supervised learning models. Notably, the signal was robust to the number of drugs involved in a combination, as we observed separation between 3-drug and 4+-drug combinations along the second PC, which was orthogonal to the first (Figure S2A).

**Figure 2:**
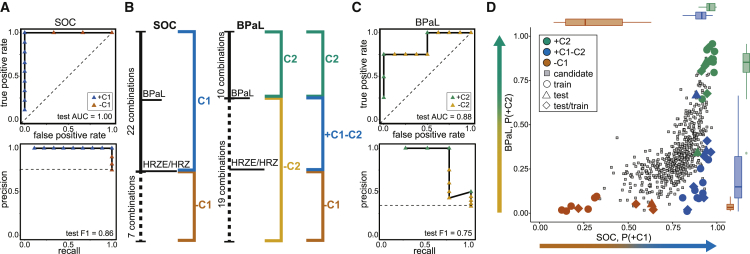
*In vitro* pairwise data are predictive of treatment improvement *in vivo* (A) Receiver operator characteristic (ROC, upper panel) and precision recall (PR, lower panel) curves associated with an SOC Random Forest classifier trained using all summary pairwise features from seven *in vitro* growth conditions. The model was trained on 70% of annotated combinations and tested on the remaining 30%. Test combinations are colored by annotation (blue = +C1, >SOC; orange = −C1, ≤SOC). (B) Schematic of combinations in the training set with annotations indicated by color and brackets. Selected combinations important for defining classes are indicated with single drug letter abbreviations (Table 1). (C) ROC and PR curves associated with a BPaL Random Forest classifier trained using all summary pairwise features from seven *in vitro* growth conditions. The model was trained and tested as in (A). Test combinations are colored by outcome annotation (green = +C2, >BPaL; yellow = −C2, ≤BPaL). (D) Probability scatterplot for SOC model predictions (P(+C1)) and BPaL model predictions (P(+C2)). Marginal boxplots show the annotated combination probability distributions. Annotated combinations are colored as in (B), and training and test combinations are labeled with circles and triangles, respectively. Combinations without annotations (candidates) are labeled with gray squares.

PCA revealed partial separation of −C1 and +C1 combinations, but the remaining overlap hinders accurate classification of candidate combinations using PCs alone. To increase classification accuracy, we turned to supervised ML. We evaluated seven ML algorithms for their ability to distinguish +C1 and −C1 combinations and compared their performance with repeated random partitioning of data for model training and evaluation. We observed ensemble methods, such as Random Forest (RF), to be top performers among the seven algorithms (Data S3), with corresponding classifiers achieving high (AUC > 0.86) accuracy on both training and test data. We, therefore, chose RF for all subsequent analyses. The representative model training and evaluation partition (see STAR Methods) included the three-drug SOC (HRZ) in the model training and performed well when evaluated on the set that included the four-drug SOC (HRZE; AUC = 1.00; Figure 2A). We retrained a model with both HRZ and HRZE withheld for model evaluation and found comparable model performance (AUC = 1.00; Figure S2B), indicating that the correct identification of combinations did not depend on the inclusion of SOC combinations in model training. To assess whether the performance of high-order drug combinations is primarily driven by the performance of single drug pairs, we compared the predicted probability of being +C1 for each combination with the highest predicted probability among the corresponding constituent drug pairs (Figure S2C). The observed trend indicates that predicted combination efficacy is not driven solely by the best-performing drug pair. Together, these results support using *in vitro* drug-pair measurements to predict improvement over the SOC in high-order combinations and suggest that an effective drug pair may be the backbone of an effective high-order combination on which more drugs can be added.

**Table 1:** Abbreviations used in this study and brief descriptions of these abbreviations

Drugs (abbreviations used in combination names)	Descriptions of abbreviations
B	bedaquiline, ATP synthesis inhibitor
C	clofazimine, antimycobacterial/multi-process inhibitor
E	ethambutol, cell wall synthesis inhibitor
H	isoniazid, cell wall synthesis inhibitor
L	linezolid, protein synthesis inhibitor
M	moxifloxacin, DNA synthesis inhibitor
Pa	pretomanid, cell wall synthesis inhibitor/nitric oxide production
Z	pyrazinamide, antimycobacterial/multi-process inhibitor
R	rifampicin, transcriptional inhibitor
P	rifapentine, transcriptional inhibitor
Su	sutezolid, protein synthesis inhibitor
Sq	SQ109, multi-process inhibitor
Treatment outcome and classification	
+C1	better than standard of care
−C1	as good or worse than the standard of care (HRZE or HRZ)
+C2	better than BPaL
−C2	as good or worse than BPaL
+C1−C2 (+C1 and −C2)	better than standard of care and worse than BPaL
SOC	standard of care
TTP	time to culture positivity
Mouse model	
RMM	relapsing mouse model
*In vitro* models	
a	acidic
b	butyrate
c	cholesterol (0.05 mM)
d	dormancy
h	cholesterol-high (0.2 mM)
s	standard
v	valerate
Data and metrics	
C	constant time point
T	terminal time point
CT	constant and terminal time points are the same
log_2_FIC_n_	fractional inhibitory concentration at n % growth inhibition
AUC_25_	the normalized area under the dose-response curve up to the 25% growth inhibitory concentration [IC_25_]
E_inf_	the effect at infinite drug concentration (maximum achievable effect)
GR_inf_	normalized growth inhibition effect at infinite drug concentration (maximum achievable effect)
ROC	receiver operator characteristic
AUC	area under the ROC curve
PR	precision recall
F1	harmonic mean of the precision and recall
Machine learning acronyms	
PC	principal component
PCA	PC analysis
RF	random forest
DT	decision tree

By necessity, TB drug regimen development is iterative in that drugs are added to or substituted into effective combination scaffolds. Testing of combinations often begins by adding or substituting a drug into a combination that has been previously tested. To simulate the process of combining a new drug with existing drugs, we treated each drug from the 12-drug dataset as a “new” drug. For this analysis, each drug was individually left out except for R, which could not be left out because too few −C1 combinations remained in the training set. We reserved combinations containing the candidate drug for testing (“leave-one-drug-out”) and trained a model on the remaining drug combinations. For each of the 11 “leave-one-drug-out” training/test sets, we included the HRZE combination (four-drug SOC) in the test set to evaluate combination prediction compared with the SOC. The models correctly predicted whether including the “left-out” drug improved treatment outcome (mean AUC ± SEM, 0.91 ± 0.04; Data S3). Although the inclusion of any one drug into the scaffold was not required for accurate performance, the exception was the model trained after B was left out, which produced a random classifier (AUC = 0.58; Data S3). Together, these results demonstrate that RMM outcomes of combinations containing a previously untested drug can be effectively predicted using pairwise *in vitro* measurements with minor drug-specific limitations.

### Additional classifiers predict top-performing combination outcomes *in vivo*

Of all possible 575 three- and four-drug combinations among the 12 drugs, only 39 (∼7%) were annotated with an RMM outcome, which we further split into 29 training and 10 test combinations. Given the SOC classifier (Figure 2A), we used the 29 annotated combinations in the training set to compute the optimal classification threshold (Youden’s J; P(+C1) = 0.71) and applied it to categorize *in vitro* data from the 10 test combinations and the remaining 536 (∼93%) candidate combinations (Data S3). We note that 76% (31/41) of the binned combinations are annotated to be +C1 (Data S3), indicating that there are likely to be many combinations that improve outcome over the SOC in the RMM. Of the 536 candidates, the classifier predicted 400 (76%) to be an improvement over the SOC, consistent with the percentage of +C1 annotated combinations from *in vivo* studies, but which is too high for effective follow-up. Selection of combinations using alternative criteria, such as the top 10% (or fewer) of predicted combinations, can aid in prioritizing combinations for *in vitro* experiments (e.g., direct high-order measurements) and *in vivo* studies (such as pharmacokinetic/pharmacodynamic [PK/PD] studies and dose optimization). We note that HRZE (part of the model test set) would place in the bottom 15% of combinations evaluated in this study (P(+C1) = 0.633; Data S3). BPaL is established as better than the SOC for both decreasing disease relapse and shortening treatment time of mice infected with drug-sensitive *M. tuberculosis*.^6^^,^^16^^,^^17^ In addition, the use of BPaL has dramatically shortened the treatment time of MDR-TB in the clinic.^5^ We, therefore, chose BPaL as a benchmark for further treatment improvement over the SOC in the RMM, despite not yet knowing the outcome of BPaL over the SOC in clinical trials for drug-sensitive TB (DS TB) treatment.

We reannotated the RMM outcome (Figure 2B) according to whether it was better than BPaL (+C2) or not (−C2; Data S2). The +C2 group is a proper subset of the SOC + C1, and the new −C2 class combines the remaining +C1 (now labeled +C1−C2) and the previously labeled −C1 combinations. As with SOC, *in vitro* pairwise data are separated by the +C2 and −C2 labels along the top PC (Figure S2D).

Using the same validation process we performed with SOC classifiers, we evaluated the performance of a model trained with features from all conditions for its ability to distinguish +C2/−C2 combinations. We observed comparable performance for the all-condition model during model training (AUC = 0.83) and high performance using the held-out test set (AUC = 0.89; Figure 2C). Model performance was relatively invariant to the measurement time points (Figures S2E and S2F). We also observed that combinations predicted to be better than BPaL (Youden’s J; P(+C2) > 0.36) also tend to have the highest likelihood to improve treatment outcome over the SOC (P(+C1) > 0.78; Figure 2D; Data S3). However, the converse is not true: high probability +C1 (P(+C1) > 0.71) combinations may or may not be better than BPaL. This suggests that the SOC and BPaL classifiers are non-redundant, and classification for improvement over BPaL (182 combinations, 34%; Data S3) can further refine the set of +C1 combinations for experimental follow-up. We observed a wide range of probabilities for the BPaL classification (P(+C2) between ∼0.35 and ∼0.75) in which there are few annotated combinations; therefore, further prioritization may be achieved using a more conservative BPaL classification threshold (e.g., P(+C2) = 0.5, 14% (73) +C2 combinations) or by ranking candidate combinations using probabilities (Figure 2D). Whichever method is used for candidate prioritization, it is important to recognize that there may exist many potential treatment-improving combinations using existing anti-TB drugs, and that prioritization schemes enable us to focus on the most promising ones. In that light, +C2 combinations represent a unique subset of potentially treatment-improving combinations.

### RMM outcome prediction is improved using subsets of *in vitro* conditions

*M. tuberculosis* encounters many environments during infection, and some are thought to contribute more than others to the requirement for long treatments. We asked which of the seven *in vitro* models were most predictive and whether a smaller set of *in vitro* conditions could be used to model RMM outcomes. We observed that data from dormancy, valerate, and butyrate conditions produced the top-performing single *in vitro* condition models for both the SOC and BPaL outcomes (Figure S3A). Models constructed from multiple conditions as a “sum-of-parts” are likely to be the most predictive because they represent different aspects of the diverse microenvironments encountered during an infection.^7^ We reasoned that a model trained with three conditions would balance the economy of the experimental scale and capture the complexity in the microenvironment and dependency of drug efficacy on those environments. Adding conditions beyond three may help refine models but did not generally improve performance (Figure S3A). Therefore, we focused our analyses on three-condition models.

**Figure 3:**
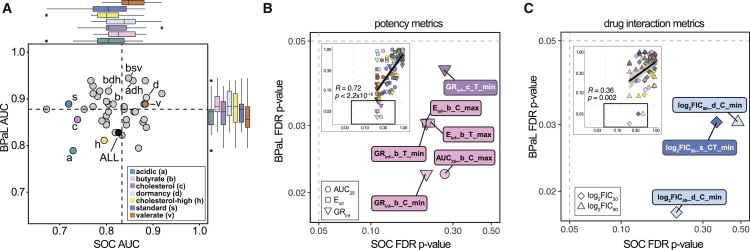
Predictive information in subsets of *in vitro* conditions and dose-response metrics (A) Scatterplot of model performance (AUC) for the SOC and BPaL machine learning models trained on data from one, three, or all (seven) conditions and evaluated in cross-validation (see Figure S3A for performance of models from any number of conditions). Marginal boxplots indicate the performance of models containing each condition. Dashed line indicates the median performance across all models. Single-condition abbreviations are as in Table 1 and the legend. (B) Scatterplot of p values from the Wilcoxon rank-sum tests contrasting values of individual potency features across SOC (−C1 versus +C1) and BPaL (−C2 versus +C2) outcomes showing features with p value < 0.05 for BPaL outcome comparison. Inset: scatterplot of all features with the region containing p < 0.05 shown in solid black rectangle. Features are colored by *in vitro* condition and shaped by metric type (circle, AUC_25_; square, E_inf_; downward triangle, GR_inf_). p values are corrected for multiple hypothesis testing (FDR) within each outcome group (e.g., corrected for SOC comparison separate from BPaL comparison). Features with FDR p < 0.05 are annotated with extra information such as time (C or T for constant or terminal, respectively) and the summary statistic type (minimum, mean, or maximum). Linear regression line (solid black), confidence interval (shaded region), Pearson correlation coefficient (R), and associated p value are indicated on plot. (C) Scatterplot of p values from the Wilcoxon rank-sum tests contrasting values of individual drug interaction features (plot elements as in B). Features are shaped by metric type (triangle, log_2_FIC_90_; diamond, log_2_FIC_50_).

After evaluating all possible three-condition models, we observed that all but one model was high performing for both outcomes (AUC > 0.7; Figure 3A), and that many performed better than the seven-condition model (SOC AUC = 0.83, BPaL AUC = 0.83). These results demonstrate that three conditions were sufficient to train models that were as good or better than a model trained on all possible condition information. We confirmed the high performance of three-condition models using test data and predicted candidate combination classification comparable with the all-condition model (Figures S3B–S3G). Furthermore, high-performing models can be trained using many aggregated sets of three conditions (Figures S3A–S3G). Finally, the high performance of three-condition sets for both BPaL and SOC outcome models suggests that using one of the two is sufficient for classifier evaluation. Therefore, we focused on only BPaL outcome models in subsequent analyses.

Conditions with important information for predicting *in vivo* outcomes should be those that improve model performance when included, even if the condition alone is not the highest performing. Therefore, we compared model training performance with and without each of the seven conditions. We expected that if a condition is sufficiently informative, a majority (>50%) of the models including it should have increased performance compared with when that condition is excluded. We observed that 65% of models saw an increase in AUC when butyrate was included, with similar trends for dormancy (54% of models) and cholesterol-high (51% of models; Figure S3H). The trend toward increased performance was maintained among models using data from four or more conditions that included butyrate + dormancy + cholesterol-high compared with those with only two or fewer of these conditions (p = 0.206; Figure S3I). Lastly, we observed that the model butyrate + dormancy + cholesterol-high was the sixth-highest-performing three-condition model for the BPaL outcome (AUC = 0.92, F1 = 0.81; Figure 3A; Data S3), and the top five three-condition models included at least one of these three conditions.

Although dormancy and butyrate were the top two highest-performing single-condition models (Data S3), the cholesterol-high condition performed modestly as a single-condition model compared with other growth environments. Nevertheless, models with other conditions improved on the addition of cholesterol-high measurements (Figure S3H), suggesting that the condition carries an orthogonal signal to other conditions.

This analysis demonstrates that there is predictive information in many of the *in vitro* models, with some conditions carrying redundant information, while others provide an orthogonal signal that improves classifier performance. Future work to prioritize combination therapies based on pairwise measurement will therefore not require exhaustive measurement in many growth conditions but can instead focus on small (two or more) *in vitro* models with established predictive accuracy.

### Treatment outcome is driven by exceptional drug pairs rather than averaged pairwise properties

The RF models classified some of the top +C1 combinations as +C2 and others as −C2 (Figure 2D), suggesting that different *in vitro* metrics were important to distinguish +C2 combinations from those used to distinguish +C1 combinations. In other words, the +C2 combinations were not simply the highest probability +C1 combinations. We sought to understand what features could accurately distinguish +C2/−C2 and +C1/−C1 combinations and what feature values constituted +C2 combinations. Therefore, we compared the statistical significance of individual metrics to distinguish SOC (+C1 from −C1) and BPaL outcomes (+C2 from −C2) using the Wilcoxon rank-sum test. We examined the values of individual features among all conditions and found that several correlated with the +C2/−C2 outcome class (9 of the 186 [∼5%] features; p < 0.05, Wilcoxon rank-sum test, using Benjamini-Hochberg multiple hypothesis correction; Figures 3B and 3C; Data S4; Figure S4A). Although no features differed significantly between +C1 and −C1 drug combinations (all p > 0.05), we nevertheless observed a strong correlation between the significance of potency features in the SOC and BPaL comparisons (Figure 3B; Pearson correlation, R = 0.72, p < 0.001). In contrast, drug interaction metric correlation between the SOC and BPaL outcome thresholds was substantially weaker (Figure 3C; Pearson correlation, R = 0.36, p = 0.002). As with potency features, several drug interaction features differed significantly between +C2 and −C2 combinations, but not between +C1 and –C1 ones (Figure 3C); this is consistent with a prior study where we found that RMM outcomes relative to the SOC were predicted by potency metrics rather than synergies.^7^

We noted that many significant features were from the butyrate and dormancy conditions (Figures 3B and 3C), supporting the use of a three-condition model including these conditions. We also observed that all the significant features to correlate with the +C2/−C2 dichotomy describe the most potent and most synergistic pairs (e.g., minimum GR_inf_ and log_2_FIC values and maximum E_inf_ and AUC_25_ values among the underlying pairs of a high-order combination). These results suggest that a small number of strong drug pairs contribute more information about treatment improvement of a high-order combination than the average behavior of all involved pairs. Furthermore, these observations are not specific to the training set and generalize when test combinations were also considered (Figures S4B and S4C). These results indicate that the degree of treatment improvement of a drug combination (over BPaL and SOC) can be predicted using *in vitro* measurements of pairwise drug potency and that there are drug-pair synergies when *M. tuberculosis* are dormant that distinguish drug combinations that are better than BPaL.

### Design principles for constructing effective drug combinations

Given that highly effective drug pairs appear to drive the treatment outcome of high-order drug combinations (Figure 3), we aimed to understand how to identify and compile effective drug pairs using *in vitro* measurements. Our goal was to compose a set of rules to guide the rational design of high-order drug combinations using drug pairs as the building blocks.

**Figure 4:**
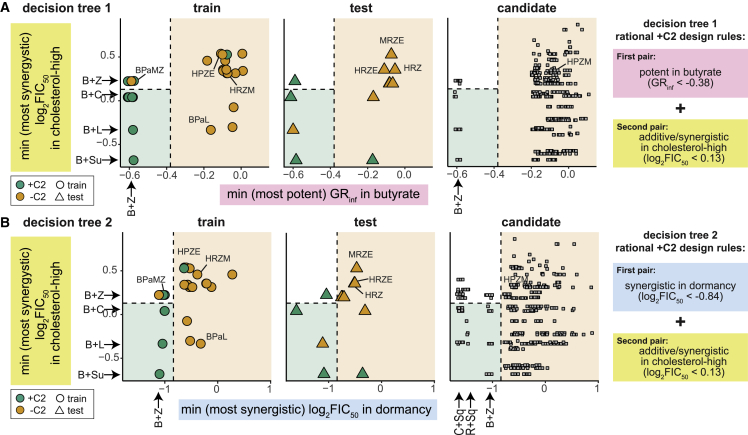
Rulesets for assembling +C2 (RMM) drug combinations based on effective drug pairs (A and B) Scatterplots of two metrics identified to be important for outperforming BPaL, shown as decision tree 1 (A) and an alternative decision tree 2 (B). Combinations are colored by annotations (green = +C2, orange = −C2). Combinations are plotted separately based on whether they were used in decision tree model training (circle, left), testing (triangle, middle), or are candidates (square, right). Selected drug combinations are indicated with labels. Regions of the plot are colored based on the decision tree classification using thresholds (dashed lines) learned during training. White region denotes satisfying rule one, but not rule two, criteria for +C2 classification. Metric values of selected drug pairs are indicated along plot margins. Rules are written in logic format on the right.

Decision tree (DT) classification mirrors human decision-making and can define a set of rules for classification tasks. To make these rules interpretable and straightforward, we focused on the features from the butyrate, dormancy, and cholesterol-high conditions, motivated by the largest increase in performance when these three conditions were included in a ML model. We selected a single-potency and drug interaction feature from each condition by choosing features with the strongest association (lowest p values) with the +C2/−C2 dichotomy based on the Wilcoxon rank-sum test analysis (Data S4). We used the training and test data split from the BPaL RF classifier and trained a DT (DT1) to identify the features and thresholds that were most informative for identifying +C2 combinations (Figure 4A). The rules defined by these features indicate that the first step in constructing a combination is to choose a potent drug pair in butyrate (GR_inf_ in butyrate at the constant time point < −0.38) and then choose a pair that is additive/synergistic in cholesterol-high (log_2_FIC_50_ in cholesterol-high at the terminal time point < 0.13; Table 2). Several candidate drug combinations were also identified using these rules as likely to be +C2. The lower complexity of two-feature DT yields did not alter accuracy when predicting the test set outcome compared with the RF classifier (83%), demonstrating that the simplicity of a short ruleset provides an accurate understanding of how to construct effective combinations based on minimal information from pairwise measurement *in vitro*.

**Table 2:** Drug pair rulesets for assembling +C2 (RMM) drug combinations

Ruleset	Pair 1	Pair 2	Figure
1	butyrate potent pair GR_inf_ < −0.38	cholesterol-high additive/synergistic pair log_2_FIC_50_ < 0.13	Figure 4A
2	dormancy synergistic pair log_2_FIC_50_ < −0.84	cholesterol-high additive/synergistic pair log_2_FIC_50_ < 0.13	Figure 4B
3	butyrate potent pair GR_inf_ < −0.38	dormancy potent pair GR_inf_ < −0.012	Figure S4D
4	butyrate potent pair GR_inf_ < −0.38	standard potent overall GR_inf_ < −0.066	Figure S4E
5	butyrate potent pair GR_inf_ < −0.38	valerate synergistic pair log_2_FIC_50_ < −0.23	Figure S4F
6	dormancy synergistic pair log_2_FIC_50_ < −0.84	acidic additive/antagonistic overall log_2_FIC_50_ > −0.062	Figure S4G
7	dormancy synergistic pair log_2_FIC_50_ < −0.84	cholesterol-high additive/synergistic drug pair log_2_FIC_50_ < 0.13	Figure S4H

The first DT (DT1) used only two features of the many that were observed to separate +C2 from −C2 combinations, suggesting that we may be able to write other rulesets. We trained a second DT (DT2), with an emphasis on dormancy and cholesterol-high features. DT2 was observed to be similar to DT1, with the second rule (of additivity/synergy in cholesterol-high) being identical. Conversely, the first decision in DT2 is based on having a highly synergistic drug pair in dormancy (Figure 4B; log_2_FIC_50_ at the constant time point < −0.84) instead of a potent pair in butyrate (from DT1; Table 2). The ability to substitute the first rule with another shows redundancies in predictive signals among the metrics in the *in vitro* dataset. Taken together, the DTs define a set of interpretable rules that can govern the rational design of effective high-order drug combinations. Notably, the rulesets are not absolute. Multiple rule variations can instruct the design of effective +C2 therapies, guided by the availability of the conditions used for the pairwise *in vitro* measurement (Table 2; for more DTs in other condition subsets, see Figures S4D–S4H).

We used DT1 and DT2 to predict classification as +C2 or −C2 on candidate combinations (Figure 4). Many drugs and drug combinations were over-represented in the combinations predicted to be +C2 (47 drugs and combinations by Fisher’s exact test, p < 0.05, after Benjamini-Hochberg multiple hypothesis correction; Data S5). Notably, we observed enrichment of combinations that include B, Z, clofazimine (C), and SQ109 (Sq), suggesting that these drugs partner well with other drugs. Prominent in these over-represented combinations is B + Z; this may be explained by how well B + Z satisfies one rule in each DT (potent in butyrate and synergistic in dormancy). However, the likelihood of high-order combinations that include B + Z to be +C2 increased when another additive or synergistic pair in cholesterol-high is also included in the combination (Figure 4A). Stated another way, if B + Z satisfied the first rule (potent in butyrate or synergistic in dormancy), a combination would be +C2 (green region) if a different pair contributed to the second rule (non-antagonism in cholesterol-high). We trained alternative DTs for other top 3 condition models (Figures S4D–S4H; Table 2). We observed that potent pairs in dormancy, butyrate, and standard medium and synergistic pairs in dormancy, cholesterol-high, and valerate are features of +C2 combinations. Because we used features that best distinguished the +C2/−C2 classification, we note that drug interactions metrics favor synergy or antagonism in a growth-condition-dependent manner (Data S4; Table 2). We also observed that a ruleset might include both synergy (a synergistic pair in dormancy) and antagonism (mean behavior of antagonism among the pairs in acidic medium) (Figure S4G; Table 2); therefore, synergy as a heuristic may be specific to the growth condition and whether a dominant drug pair or average pairwise drug interaction is considered.

We conclude that when *in vitro* pairwise data are predictive of combination treatment outcomes *in vivo*, simplified and intuitive heuristics can be developed to define and interpret design principles on how to construct combinations from the bottom up. While our RF classifiers leverage a larger dataset to provide more accurate predictions, a rules-based approach will enable us to glance at systematic pairwise drug response metrics in *M. tuberculosis* to optimize combination therapies without running classifiers (Table 2). To fully realize the potential of our drug combination dataset and aid in this “at-a-glance” approach to combination building, we have provided heatmaps of key pairwise drug combination metrics (Figure S5).

### Translation of combination drug design principles to clinical outcomes

**Figure 5:**
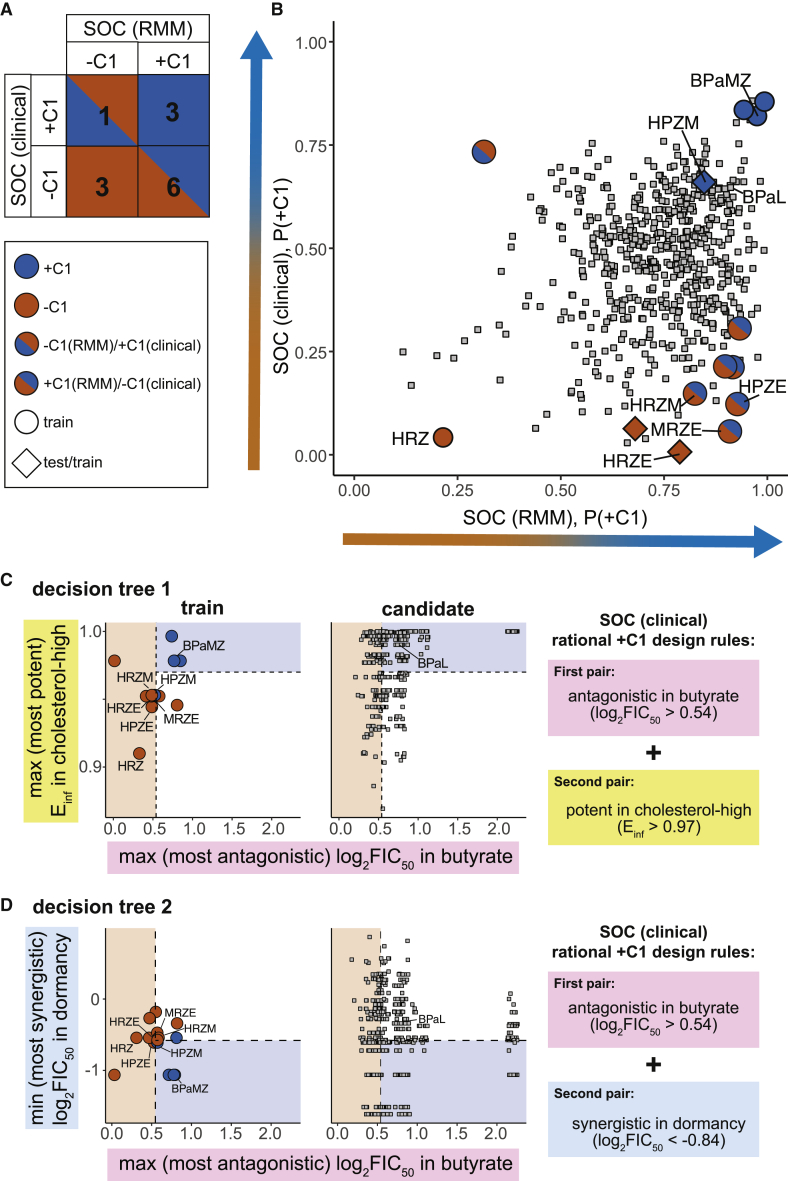
Modeling and rational design principals applied to clinical SOC outcome (A) Overlap in +C1(blue) and −C1(red) drug combination categorization between SOC (RMM) and SOC (clinical) outcomes. Blue/red squares highlight differences between outcome annotation. (B) Probability scatterplot (P(+C1)) for RMM model predictions and clinical model predictions using the butyrate + dormancy + cholesterol-high condition data. Annotated combinations are colored as in (A). Model training combinations for both RMM and clinical are labeled with circles. Combinations used for testing the RMM model and training the clinical model are labeled with diamonds. Candidate combinations (without annotations) are labeled with gray squares. (C and D) Scatterplots and alternative rulesets for two metrics identified as important for outperforming the SOC for decision tree 1 (C) and decision tree 2 (D) trained for clinical bactericidal outcomes. Combinations are plotted and shaped as in Figure 4.

The effectiveness and interpretability of the classifiers and rules for rationally designing combinations support the utility of the presented *in vitro* dataset for understanding the drivers of drug combination efficacy in preclinical mouse models. In principle, this methodology is agnostic to the *in vivo* outcomes that the models will be trained on, as long as the *in vitro* conditions are predictive of the infection-site pharmacodynamics. We next asked whether our *in vitro* data could inform our understanding of clinical outcomes of drug treatment. Bactericidal activity is the standard outcome used in phase 2 studies to evaluate treatments. We compiled a list and annotated the outcome of drug combinations evaluated for bactericidal activity in phase 2a and phase 2b clinical trials (Data S2). Clinical outcomes were scored relative to the SOC, because BPaL is not yet known to be treatment shortening relative to the SOC for DS TB in clinical studies (in contrast with the RMM, in which BPaL has been shown to improve treatment over the SOC). Consistent with previous studies,^18^, ^19^, ^20^, ^21^, ^22^, ^23^ we observed some discordance in the classification of the effectiveness of drug combinations between RMM and clinical outcomes (Figure 5A; Data S2). This discordance is expected because the outcomes (bactericidal versus relapse) are different and suggest that a model trained for SOC (RMM) may not necessarily predict combinations with bactericidal efficacy in clinical studies. Two of the six discordant combinations were HRZM and MRZE; both failed to improve HRZE in the ReMOX clinical trial^21^ and are −C1 in our clinical annotation. We previously annotated both combinations for bactericidal activity in the BALB/c mouse model as −C1,^7^ suggesting that the source of discordance may be the difference in outcome type. Due to the high cost of misidentifying combinations for follow-up in clinical trials, developing models and rules that identify potentially treatment-improving combinations in clinical trials, separate from the preclinical predictions, is highly important. Furthermore, we expect that refined prioritization of drug combinations for intensive *in vivo* and dosing studies can be achieved by combining the predictions from preclinical and clinical models.

There were too few drug combinations with clinical outcome scores to evaluate models with a held-out test set. Therefore, we trained RF classifiers using the same approach applied for SOC and BPaL RMM and assessed their performance in cross-validation. We observed high performance (AUC > 0.8, similar to performances in the RMM) in models using many subsets of conditions (Data S3). Dormancy alone was a predictive condition (AUC = 0.77; Data S3), suggesting that treating non-replicating *M. tuberculosis* is important for identifying effective drug combinations in humans. As with the BPaL predictions in the RMM, synergy in dormancy is associated with improved clinical outcomes (Data S4; log_2_FIC_50_ dormancy minimum: p = 0.03; false discovery rate [FDR]: p = 0.41). The three-condition subset of butyrate + dormancy + cholesterol-high was the highest-performing three-condition model (Data S3), suggesting that the information in these three conditions may be highly informative for understanding *in vivo* drug treatment in the BALB/c mouse model of TB, as well as in humans. Together, these results support using the *in vitro* data to train models that can be used to predict treatment outcomes in humans.

We generated predictions using the high-performing butyrate + dormancy + cholesterol-high model. We observed that candidate combinations had different predicted classifications for the SOC RMM and clinical models (Figure 5B), mirroring the discordance we previously noted (Figure 5A). Many candidate combinations were predicted to be +C1 for the clinical outcome (447, ∼79%; Figure 5B; Data S3), suggesting there may be many treatment-improving combinations remaining to be tested using existing TB drugs. We compared the BPaL (RMM) and SOC (clinical) categorization (Figure S6A) and RF classifiers and found a weak but significant correlation between their predictions (Figure S6B; Spearman’s rho = 0.31, p < 0.001). Notably, there were no combinations predicted (or annotated) to be better than BPaL in RMM that were also worse than SOC in the clinic, supporting the use of the RMM for identifying treatment-improving combinations using BPaL as a benchmark.

To define a set of rules for rationally designing clinically effective drug combinations, we used the DT approach and generated two example rulesets (Figures 5C and 5D). The clinical rulesets require antagonism in butyrate and potency in a lipid-rich environment (or synergy in dormancy; Figures 5C and 5D). Although it is not intuitive to choose combinations with antagonistic pairs, we previously identified high-order drug combination antagonism as important for classifying +C1 drug combinations,^7^ supporting the notion that in some growth conditions, average *in vitro* drug-pair antagonism may be associated with better outcomes relative to the SOC in both clinical and mouse studies. In other conditions, particularly in dormancy (Figures 3C and 5D), synergy should be prioritized. For example, we observed synergy between H and Z in dormancy (Data S1). This pair was recently observed to be synergistic in patients by PET-CT imaging in the first few weeks of clinical treatment.^24^ For the SOC (clinical) DT, we note that HPZM, the intensive phase drug combination from study 31 that shortens treatment time over the SOC in clinical trials,^4^ satisfies one rule (antagonism in butyrate) but barely misses the threshold for the second rule (potency in cholesterol-high or synergy in dormancy), incorrectly predicting it to be −C1. The HPZM DT predictions suggest that although useful for developing interpretable rules, which can guide combination design, each DT model may be sensitive to variations in individual metrics in ways that the RF classifiers are more robust. However, using either RF or DT models, we predict that there are treatment-shortening combinations among the drugs in this 12-drug set (Data S3; Figures 5C and 5D). We anticipate that as more clinical data become available, we will refine the rulesets and improve prediction accuracy. Using *in vitro* drug combination measurements, rules for rationally designing drug combinations can be written that are interpretable and allow for comparisons between human and preclinical studies.

## Discussion

Our original goal in this study was to make the prediction of *in vivo* outcomes more efficient by factoring high-order combinations into drug pairs. Classifications based on pairwise measurements increased efficiency for predicting three- and four-way drug combination outcomes in mouse models of disease relapse (RMM) by around 10-fold compared with direct DiaMOND measurement of each high-order combination. We introduced a higher threshold for classifications (better than BPaL) and predicted outcomes in 536 candidate combinations with no published RMM outcomes.

Factorization of high-order combinations into pairwise drug units enabled us to develop predictive and interpretable models. We learned that a drug pair could be a building block on which to assemble high-order combinations and defined rulesets guiding treatment-improving high-order combination construction. One such ruleset combines a drug-pair synergistic in the *in vitro* dormancy condition, with another pair potent in a high-cholesterol growth medium. Although our ML models are more accurate, these simple rules enabled rational combination design. We found that the principles of combination design in the RMM translate to clinical outcomes. For example, the clinical and RMM rulesets included pairs that are synergistic in dormancy and potent in lipid-rich conditions. This is intuitive and consistent with the notion that improved treatments will target the most refractory bacteria (dormant) in an infection and aligns with the decades of preclinical and clinical studies.^2^^,^^25^, ^26^, ^27^ Constructing combinations from building-block pairs is harmonious with the approaches currently used to design preclinical studies and clinical trials; an effective base combination is augmented by addition of drugs.^2^^,^^18^^,^^21^^,^^28^, ^29^, ^30^, ^31^, ^32^, ^33^, ^34^, ^35^, ^36^ Using the available data, we predict there are treatment-improving combinations in the existing drug combination space. The design principles presented here will allow candidate combination construction “at a glance” using cost-effect pairwise combination measurement.

The rulesets we define establish a framework for combination design in experimentally tractable sizes: properties of a drug pair. We anticipated averaged pairwise data to predict combination outcome. Instead, properties defining the “best” (e.g., most potent or most interacting) pair in a combination were most informative. Ideally, each objective in a ruleset should be achieved with a different pair. In this way, each pair can be viewed as a building block that not only enables us to construct combinations rationally but also to identify how established combinations may be improved. Our initial rulesets assemble two pairs into three-way combinations but generally leave a degree of freedom for choosing a fourth drug. We expect to define the third rule and enable four-drug combination design when more four-way *in vivo* data are available for model training.

The features used in each rule are also specific to the metric type, e.g., potency or interaction, allowing us to evaluate whether synergy is a requirement of the best combinations. We found synergy separates combinations at the BPaL threshold, especially in dormancy. Classification around the SOC is not driven by pairwise (this study) or high-order^7^ drug interactions. Furthermore, antagonism (not synergy) in *in vitro* models such as butyrate increases the likelihood of a combination performing better than the SOC *in vivo*. These seemingly disparate rules may reflect the difference between achieving treatment efficacy (SOC) and improving treatment (better than BPaL) or may be indicative of which populations are easiest to sterilize (potent drug pairs kill actively growing cells) compared with those where synergy is necessary (dormancy). Drugs that enhance the effect of each other could aid in targeting the most refractory cells in an infection (e.g., dormant/non-replicating).^2^^,^^25^, ^26^, ^27^^,^^37^^,^^38^ Further study is required to evaluate where these rulesets can be understood in the drug response in granulomas where *M. tuberculosis* residing in multiple compartments can respond differentially to treatment.

TB drug regimen design is a lengthy, multi-step process, including drug discovery, *in vivo* testing in progressively intensive animal models, toxicity testing, dose optimization, and clinical trials. Tens (but not thousands) of combinations will be progressed for *in vivo* and optimization studies. We envision the experimental and computational framework devised in this study, supported by other evaluation methods, can be used early in regimen development to winnow down the thousands of potential combinations to a priority list for further evaluation using PK/PD studies and animal testing (Figure S1).

### Limitations of the study

Predictive models are only as good as the data on which they are based, so our ability to make predictions and interpret combination design principles are dependent on the availability of *in vivo* and clinical combination data. We anticipate refining and improving our classifiers with incorporation of new clinical trial data and *in vivo* tests of our predictions and combinations including antibiotics with new target profiles.^3^ To accommodate many possible drug combinations early in drug development, our analyses do not account for differences in drug access sites, infection sites, or doses. Incorporating dosing and pharmacokinetics may improve the predictive ability of models against clinical outcomes. Together, the iterative modeling and systematic measurement of pairwise drug combinations in validated *in vitro* conditions will allow us to use best the rich information provided by preclinical and clinical studies through parallel *in vitro* studies, making bottom-up and top-down coordinated methods for the rational design of combination therapies for TB.

## STAR★Methods

### Key resources table

**Table:** 

REAGENT or RESOURCE	SOURCE	IDENTIFIER
**Bacterial and virus strains**		

*Mycobacterium tuberculosis*: Strain Erdman + pMV306hsp + LuxG13	^7^	N/A

**Chemicals, peptides, and recombinant proteins**		

Bedaquiline	NIH AIDS Reagent Program	N/A
Clofazimine	Sigma	C8895
Ethambutol	Sigma	E4630
Isoniazid	Sigma	I3377
Linezolid	Sigma	PZ0014
Moxifloxacin	Sigma	SML1581
Pretomanid	TB Alliance	N/A
Pyrazinamide	Sigma	PHR1576
Rifampicin	Sigma	R3501
Rifapentine	Sigma	R0533
Sutezolid	Sigma	PZ0035
SQ109	Sequella, Inc	N/A

**Deposited data**		

Drug pair measurements for 10 drugs	^7^	N/A
Drug pair measurements for SQ109	^14^	N/A
Drug pair measurements for SQ109 and SUT	This paper	Data S1
Code for modeling	This paper	Data S6

**Software and algorithms**		

Code for modeling, and figure generation	This study	Data S6
MATLAB	The Mathworks Inc.	https://www.mathworks.com/products/matlab.html; RRID:SCR_001622
R Project for Statistical Computing	R Foundation for Statistical Computing	https://www.R-project.org/; RRID:SCR_001905
tidyverse(R package)	^39^	https://CRAN.R-project.org/package=tidyverse; RRID:SCR_019186
ggplot2(R package)	^40^	https://CRAN.R-project.org/package=ggplot2; RRID:SCR_014601
ggpubr(R package)	N/A	https://CRAN.R-project.org/package=ggpubr; RRID:SCR_021139
Ggrepel	N/A	https://CRAN.R-project.org/package=ggrepel; RRID:SCR_017393
openxlsx(R package)	N/A	https://CRAN.R-project.org/package=openxlsx; RRID:SCR_019185
readxls(R package)	N/A	https://CRAN.R-project.org/package=readxl; RRID:SCR_018083
stats(R package)	N/A	https://www.R-project.org/
paran(R package)	N/A	https://CRAN.R-project.org/package=paran
mlr(R package)	^41^	https://CRAN.R-project.org/package=mlr
bartMachine(R package)	^42^	https://CRAN.R-project.org/package=bartMachine
randomForestSRC(R package)	^43^	https://CRAN.R-project.org/package=randomForestSRC
xgboost(R package)	^44^	https://CRAN.R-project.org/package=xgboost
e1071(R package)	N/A	https://CRAN.R-project.org/package=e1071
kknn(R package)	N/A	https://CRAN.R-project.org/package=kknn
rstatix (R package)	N/A	https://CRAN.R-project.org/package=rstatix; RRID:SCR_021240
wPerm (R package)	N/A	https://CRAN.R-project.org/package=wPerm
Python	python	https://www.python.org/; RRID:SCR_008394
Xlrd (python package)	N/A	https://xlrd.readthedocs.io/en/latest/#; RRID:SCR_022257
Pandas (python package)	^45^	https://doi.org/10.5281/zenodo.3509134; RRID:SCR_018214
Matplotlib (python package)	^46^	https://zenodo.org/record/1420605#.Yu5JhezMLlw; RRID:SCR_008624

### Resource availability

#### Lead contact

Further information and requests for resources and reagents should be directed to and will be fulfilled by the lead contact, Bree Aldridge (bree.aldridge@tufts.edu).

#### Materials availability

This study did not generate new unique reagents.

### Experimental model and subject details

#### Bacterial cell lines and culture

A previously transformed autoluminescent strain of the *M*. *tuberculosis* Erdman strain was used for all experiments in this study.^7^ Mtb was maintained using a standard 7H9 Middlebrook medium supplemented with 0.2% glycerol, 10% OADC (0.5 g/L oleic acid, 50 g/L albumin, 20 g/L dextrose and 0.04 g/L catalase), 0.05% Tween-80, and kanamycin (25 μg/mL). Unless noted, all culturing was performed at 37°C with aeration. Cell passaging was performed before reaching OD_600_ = 0.7.

### Method details

#### In vitro *pairwise drug response measurements*

To expand the pairwise drug combination response dataset from 10-drugs^7^ to 12-drugs, we used DiaMOND to measure 2-way dose-response curves with sutezolid and SQ109 against each other and the 10-drug set. The pairwise data with sutezolid is new to this study. Some of the SQ109 pairwise measures were reported,^14^ and the remaining combination measures in other growth environments are new to this study. All experiments were performed using the same procedures previously described.^7^ Briefly, drug response was measured using an autoluminescent reporter strain of *M*. *tuberculosis* Erdman^7^ (transformed with a single copy chromosomal integration of pMV306hsp + LuxG13^47^), and metrics were averages of at least biological duplicate experiments. DiaMOND requires single- and equipotent drug combination dose responses to determine the potency and drug interactions. A 1.5-fold, ten-dose resolution dose-response was used for all experiments. SQ109 and sutezolid (non-metabolite form) were provided by Sequella, Inc. Drugs were stored and dispensed in DMSO using an HPD300e digital drug dispenser.

The base medium of the standard and acidic *in vitro* models consisted of 7H9 Middlebrook medium supplemented with 10% OADC (0.5 g/L oleic acid, 50 g/L albumin, 20 g/L dextrose, and 0.04 g/L catalase), 0.05% Tween-80, and 25 μg/mL kanamycin (to maintain selection of reporter-carrying Mtb). The base medium of the other *in vitro* models was 7H9 (4.73 g/L) supplemented with fatty acid-free BSA (0.5 g/L), NaCl (100mM), tyloxapol (0.05%), and 25 μg/mL kanamycin. All *in vitro* model media were buffered to pH7.0 with 100 mM MOPS except acidic (buffered to pH5.7 with 100 mM MES). Carbon sources were added to *in vitro* model media to final concentration as follows: acidic and standard (glycerol, 0.2%), butyrate and dormancy (sodium butyrate, 5mM), valerate (valeric acid, 0.1%), cholesterol (cholesterol, 0.05mM), and cholesterol-high (cholesterol, 0.2 mM).

Mtb were acclimated to *in vitro* model growth medium for 2–6 doubling times prior to treatment for the DiaMOND assays. Doubling time in days were previously determined and are as follows: standard (0.8), acidic (2), butyrate (2), valerate (3), cholesterol-high (4), cholesterol (7). Acclimated Mtb were seeded at OD_600_ = 0.05 at 50uL per well into 384-well plates with antibiotics pre-dispensed. The simple dormancy model is based on the butyrate medium, supplemented with sodium nitrate (5mM), sealed, and cultured without aeration to lower oxygen levels. After 28 days, these non-replicating Mtb are plated (20uL per well) on antibiotic-seeded wells, the plates sealed and incubated. After seven days, 80uL of standard medium was added to each well, and plates were incubated with aeration for recovery and growth inhibition measurements.

Growth inhibition was measured by OD_600_ (for all conditions except dormancy) or luminescence (dormancy) using a Synergy Neo2 Hybrid Multi-Mode Reader. The constant and terminal times are as follows in days, respectively: standard (4.2), acidic (6, 12), butyrate (6, 10), valerate (9, 15), cholesterol-high (12, 24), cholesterol (7, 28), dormancy (2, 4 into recovery). Growth inhibition measurements were processed, and dose-response metrics were calculated using custom scripts written in MATLAB.

#### Drug pair dataset and data structure

For modeling and analysis, we used a 12-drug, 2-way drug response dataset comprised of data from a 10-drug combination dose-response DiaMOND dataset,^7^ a DiaMOND study of drug interactions with SQ109,^14^ and new measurements (sutezolid combinations and select SQ109 combinations). Drug pair data were selected from the datasets and used the dose-response metrics (AUC_25_, E_inf_, GR_inf_; higher (positive) AUC_25_ and E_inf_ values are potent and lower (negative) GR_inf_ values are potent) and drug interactions (log_2_FIC_50_, log_2_FIC_90_; negative and positive values indicate synergy and antagonism, respectively) from the constant and terminal time points. These drug pair metrics were aggregated via the minimum, maximum, and mean summary statistics for each high-order drug combination. Drugs with the same mechanism of action were excluded from any drug pair and high-order drug analysis (i.e., linezolid + sutezolid or rifampicin + rifapentine are not in candidate combinations).

#### in vivo *annotation of drug combinations*

Comparing drug treatment outcomes between studies necessitated an annotation scheme relatively insensitive to differences in study methodologies, including infection inoculum, drug dosing, treatment time, and Mtb strain. We initially chose comparison to the SOC because most studies include the SOC treatment for evaluating drug treatment outcomes and this comparison is generally accepted as a benchmark to determine if a drug combination should continue to be followed up. Annotations of drug combinations for the SOC outcome (+C1/-C1) were taken from a previous study.^7^ In brief, combinations with lower relapse (increased efficacy), similar relapse percentage with shorter treatment time (treatment shortening), or both, over the SOC were annotated as + C1. Combinations with equivalent or worse outcomes by these criteria compared with the SOC were annotated as -C1. Combinations in studies including SOC treatments enabled direct annotation. When no SOC treatment was included in a study, an inferred combination annotation was attempted by using a combination from the study that was annotated in a separate, direct comparison to SOC study as a cross-study reference. If a combination was tested at multiple doses, the most efficacious dose was used for annotation. Combinations remained unannotated if no direct or inferred comparison to SOC could be made. Combinations tested at multiple doses were annotated based on the best performing dose. The same studies and annotation strategy were used to annotate the BPaL outcome (+C2/-C2) with BPaL as the benchmark instead of the SOC.

Clinical studies evaluating the bactericidal activity of drug combinations using culture negativity or time to positive (TTP) at different intervals during treatment were annotated as described above (Data S3). The conclusions about differences in drugs and combinations effects were shown to be smaller but comparable for many treatments at 14 days (outcome in phase 2a trials) as compared to 56 days (outcome in phase 2b trials).^48^^,^^49^ Twelve combinations were evaluated in Phase 2b trials for bactericidal activity using either culture negativity or TTP culture microbiological outcomes after eight weeks of treatment. To increase the number of combinations for training machine learning models and because of the high clinical efficacy of bedaquiline-containing combinations, we also included one Phase 2a study,^50^ where three bedaquiline-containing combinations (B + C + Pa + Z, B + C + Pa, B + C + Z) were tested for early bactericidal activity after 14 days of drug treatment using the TTP outcome. We confirmed that including these combinations did not skew our candidate prediction results by comparing predictions to those made by a model that excluded these three combinations (R = 0.96 Pearson correlation, Figure S7).

#### Data processing, analyses, and visualization

All data processing, computational analyses, and visualizations were performed in R (v4.0.1) using the tidyverse environment packages (v1.3.0), except heatmaps that were performed in python (v3.5). The readxls (v1.3.1) and openxlsx (v4.1.4) packages were used for data table import and export. The prcomp function from the stats package was used for PCA. Features with more than 35% missing data points were excluded from machine learning and PCA. Mean value imputation^51^ was used for the remaining features with missing data. All features were mean-centered and scaled to unit variance prior to PCA. The ggplot2 (v3.3.0), ggpubr (v0.3.0), and ggrepel (v0.9.1) packages were used for all visualizations. For heatmaps, the xlrd (v2.0.1) was used to import data, pandas (v0.24.2) for data preparation, and Matplotlib (v3.0.2) to visualize. The scripts used for the analysis and visualization of results are provided in the supplemental information (Data S6).

#### Machine learning

All machine learning tasks, including model training and evaluation in cross-validation, were performed using the “machine learning in R” (mlr v2.17.0) package with individual learners loaded from additional packages (random forest, randomForestSRC (v2.9.3); Bayesian additive regression tree, bartMachine (v1.2.6); extreme gradient boosting, xgboost, (v1.4.1.1); k-nearest neighbor, kknn (v1.3.1); logistic regression, stats (v4.0.1); naïve Bayes, naiveBayes (v0.9.7); neural net, neuralnet (v1.44.2)). Models were evaluated on a 30% proportion of data (test) withheld from training. The test/training split was selected by random 30/70% partitioning of the data ten times and identifying a representative partition that had closest estimated model performance to the mean of the ten iterations (Data S3). Where appropriate, model performance was also estimated via cross-validation with a Monte-Carlo resampling strategy that partitioned the training (70% proportion) data into further 70/30% training/test splits across ten iterations. The Youden’s J^52^ was used to select the optimal classification threshold based on training data.

#### Decision tree and ruleset determination

Decision trees were constructed in R using the rpart function (rpart package, v4.1–15), and rules and thresholds were analyzed using the rpart.plot package (v3.1.0). The minimum number of combinations for splitting a node was set to two, and the minimum terminal leaf size was set to five (RMM SOC and BPaL) or two (clinical SOC). Trees were allowed to grow fully.

#### “Leave-one-drug-out” analysis

For each of the 12 drugs, annotated combinations containing that drug were withheld from model training. Models were trained with the remaining annotated combinations, and performance on data containing the withheld drug was determined.

#### Drug pair enrichment analysis

To determine if + C2 combinations contained signature sets of drugs, we tested for over-representation in the +C2 candidate drug combinations using Fisher’s Exact Test. We performed tests for each drug, drug pair, and three-drug combination and controlled the false discovery rate (FDR).^53^

### Quantification and statistical analysis

#### Statistical analysis

Statistical analyses were performed using the stats, g1gpubr (v0.3.0), and rstatrix (v0.5.0) packages in R. Statistical significance threshold was chosen to be less than 0.05, unless otherwise indicated. The Wilcoxon rank-sum test was used to compare mean values across outcome groups. The Benjamini-Hochberg method was used to control the false discovery rate (FDR) for multiple hypothesis testing.^53^ Pearson’s correlation was used to measure linear correlations. Fisher’s Exact Test was used to test for over-representation analyses.

## Data Availability

• The published article includes all datasets generated or analyzed during this study. • All original code is available in this paper’s supplemental information. • Any additional information required to reanalyze the data reported in this paper is available from the lead contact upon request.

## Supplemental information

Document S1. Figures S1–S7

Data S1. Pairwise *in vitro* data, related to Figures 2-5, S2-S7, and Data S3-S5

Data S2. Drug combination *in vivo* outcome annotations, related to Figures 2-5, S3-S7, and Data S3-S5

Data S3. ML modeling performances and predictions, related to Figures 2-4, and Data S3-S5

Data S4. Univariate analysis of features for drug combination class separation, related to Figures 3 and S4

Data S5. Drug pair enrichment analysis, related to Figures 4 and S4

Data S6. Original code for generating figures and tables, related to Figures 2-5, S2-S7, and Data S1, S3-S5

Document S2. Article plus supplemental information
