# Supplementary material for: Design principles to assemble drug combinations for effective tuberculosis therapy using interpretable pairwise drug response measurements
Source: Cell Rep Med. 2022 Sep 8;3(9):100737. doi: 10.1016/j.xcrm.2022.100737 (PMC9512659; doi:10.1016/j.xcrm.2022.100737)
Supplement: Document S1. Figures S1–S7 [file mmc1.pdf]

Cell Reports Medicine, Volume *3*

Supplemental information

Design principles to assemble drug combinations for effective tuberculosis therapy using interpretable pairwise drug response measurements

Jonah Larkins-Ford, Yonatan N. Degefu, Nhi Van, Artem Sokolov, and Bree B. Aldridge

Supplemental Information

Figure S1

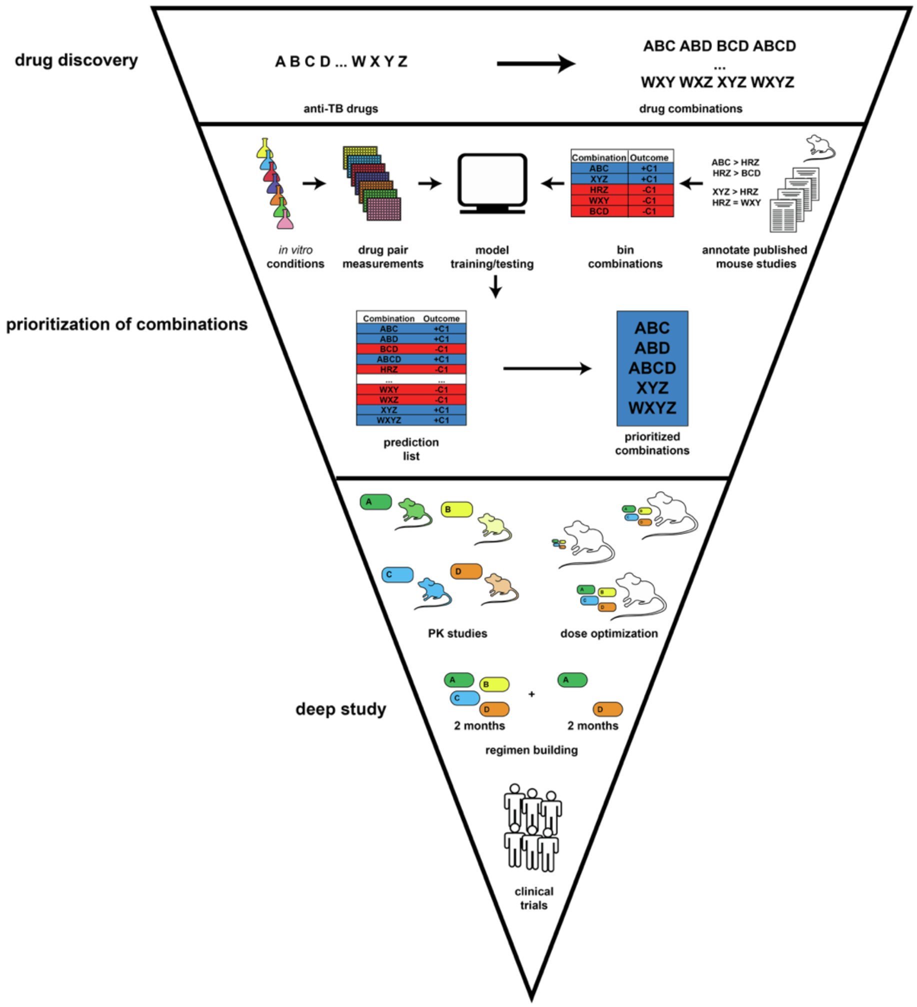

Figure S1. Anticipated positioning of pairwise modeling and predictions in the development of new TB combination treatments, related to Figure 1. Diagram of TB regimen development envisioned as a tiered pipeline from drug discovery (top tier), to prioritization of combinations using the modeling approach presented in the current manuscript (middle tier), to deep study of drug combinations, including PK studies, dose optimization, regimen design and clinical trials (bottom tier). The size of each tier indicates the relative number of drug combinations to be evaluated from the most at the top to the least at the bottom. PK, pharmacokinetic. A, B, C, D, W, X, and Y indicate hypothetical new drugs. HRZ is the three-drug SOC and +C1 is annotation for better than the SOC.

Figure S2

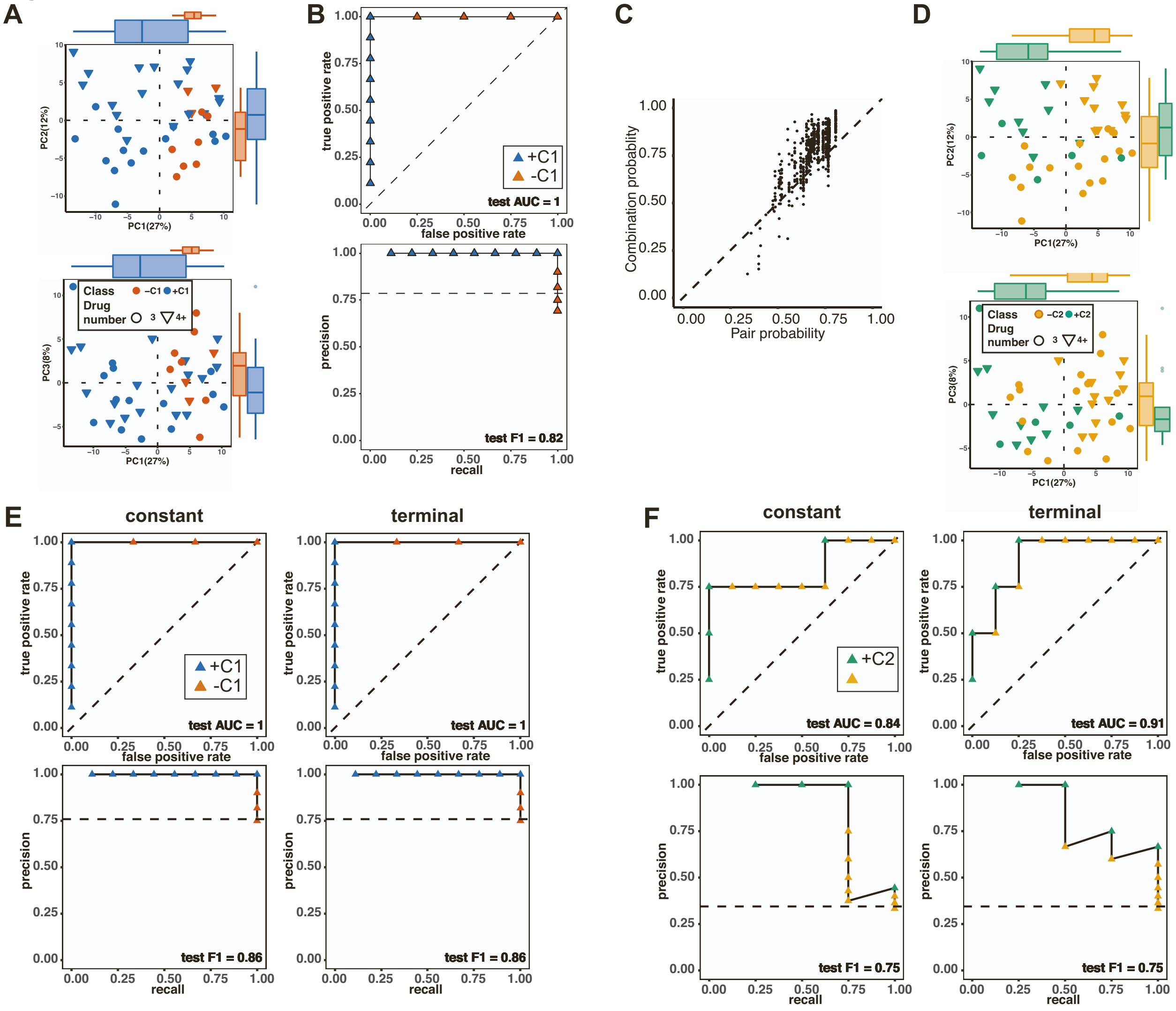

Figure S2. Pairwise metrics distinguish higher-order combination in vivo outcomes, related to Figure 2. (A) Separation of SOC annotated drug combinations by PCA. Projection of the pairwise *in vitro* combination data from all *in vitro* models onto PCs 1 and 2 (top) and PCs 1 and 3 (bottom). Points are colored by SOC outcome in the RMM: blue=+C1, better than standard of care; red=-C1, standard of care or worse. Percent variance explained by each PC indicated in the axis title. Outer box and whisker plots show the distributions of combination classes along each PC. (B) Excluding SOC from model training. ROC and PR curves associated for SOC random forest classifiers with HRZ and HRZE excluded from model training. ROC (top) and PR (bottom) curves are labeled as in Figure 2A. (C) Higher-order drug combination prediction probabilities are influenced by more than the best pair in a combination. Scatter plot of 3- and 4-drug combination prediction probabilities compared with the highest probability drug pair in each combination. Dashed line indicates the identity line where the probability is the same for both higher-order combination and drug pair. (D) Separation of BPaL annotated drug combinations by PCA. The same pairwise *in vitro* combination data projection onto PC space presented in panel A colored by BPaL outcome in the RMM: green=+C2, better than BPaL; orange=-C2, BPaL or worse. (E) and (F) Individual time-point model performance. ROC and PR curves associated with SOC random forest classifiers (E) and BPaL random forest classifiers (F) with constant time point (left) and terminal time point (right) only data used in model training and testing. ROC (top) and PR (bottom) curves are labeled as in Figure 2A.

Figure S3

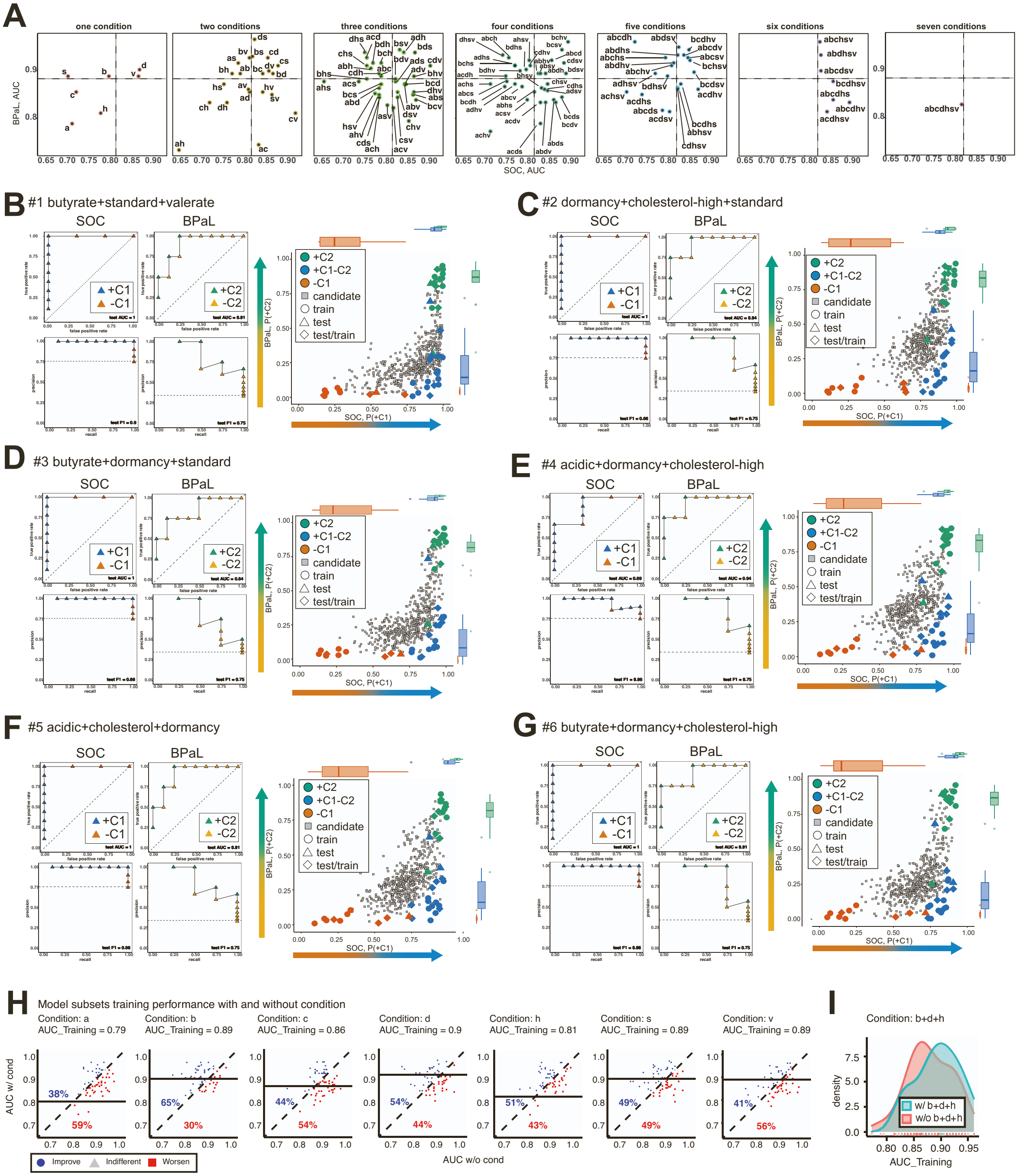

Figure S3. ML model performance using subsets of *in vitro* conditions, related to Figure 2. (A) Model performance across different numbers of *in vitro* conditions. Scatter plots of model training AUC for SOC and BPaL classifiers for models trained with data from indicated number of *in vitro* conditions (one to seven). Median performance of every model is shown with black dashed lines (SOC AUC=0.83, BPaL AUC=0.88). (B-G) Top three-condition model performance and predictions. Test performance for the six highest performing three-condition models during training: (B) butyrate+standard+valerate, (C) dormancy+cholesterol-high + standard, (D) butyrate+dormancy+standard, (E) acidic+dormancy+cholesterol-high, (F) acidic+cholesterol+dormancy, (G)

butyrate + dormancy + cholesterol-high. ROC (top) and PR (bottom) curves are labeled as in Figure 2A. Probability scatter plots are on the right and labeled as in Figure 2D. (H) and (I) Contribution of conditions to model performance. (H) Scatter plots of training performance for models without the indicated condition compared to models including the indicated condition. Change in model performance by inclusion of the condition is indicated by color (increased (blue), decreased (red), or indifferent (grey)). Dashed line indicates the line of “indifference”, where model performance does not change with or without indicated condition. Single condition training performance indicated above plot and with solid line. Percentage of models with increased or decreased performance are shown. (I) Model performance density plot of models with (green) and without (red) butyrate+dormancy+cholesterol-high (red).

Figure S4

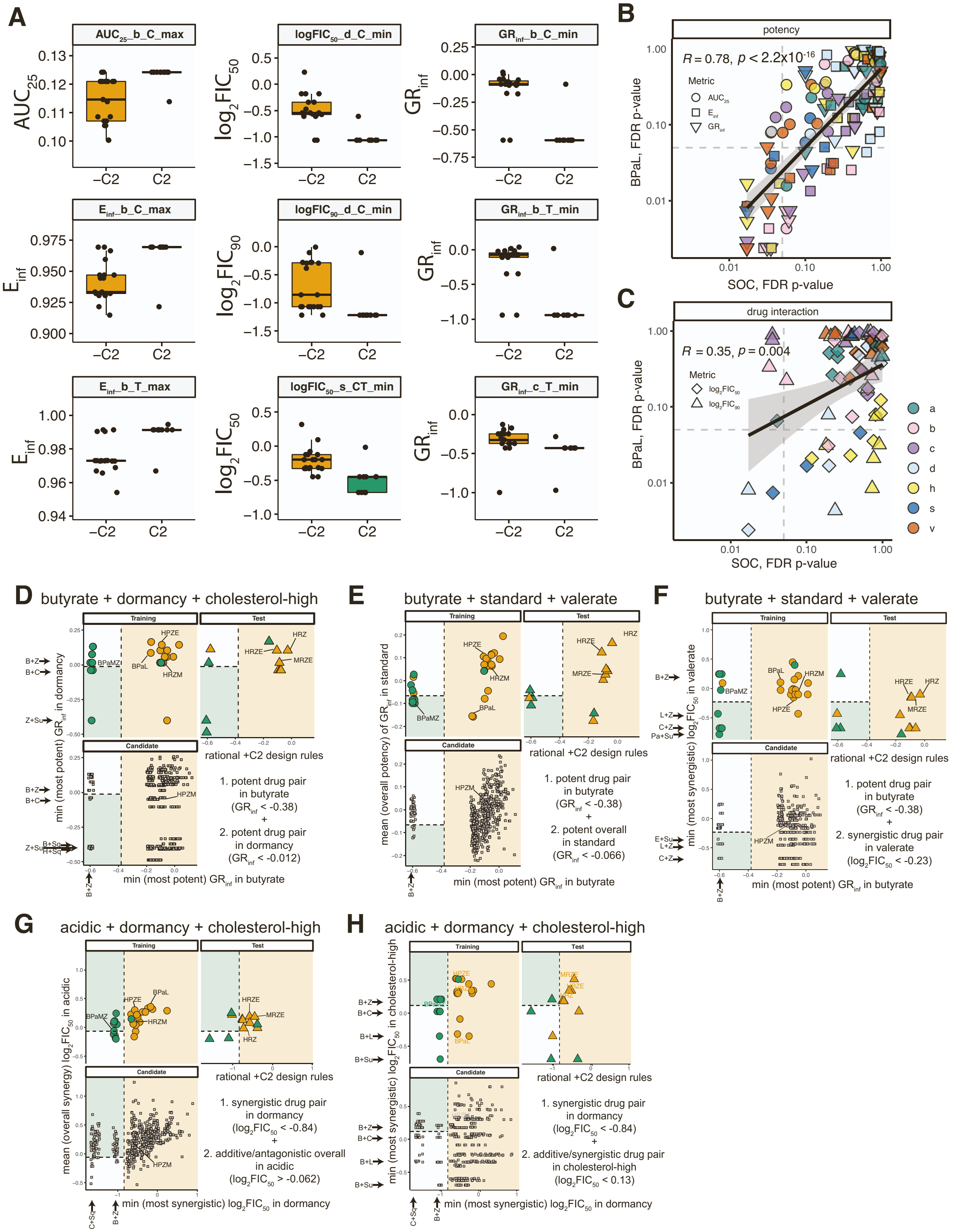

A
AUC25_b_C_max
logFIC50_d_C_min
GRinf_b_C_min
Einf_b_C_max
logFIC90_d_C_min
GRinf_b_T_min
Einf_b_T_max
logFIC50_s_CT_min
GRinf_c_T_min
–C2
C2
B
potency
R = 0.78, p < 2.2x10-16
Metric
AUC25
Einf
GRinf
BPaL, FDR p-value
SOC, FDR p-value
C
drug interaction
R = 0.35, p = 0.004
Metric
log2FIC50
log2FIC90
a
b
c
d
h
s
v
D
butyrate + dormancy + cholesterol-high
Training
Test
Candidate
rational +C2 design rules
1. potent drug pair in butyrate (GRinf < -0.38)
+
2. potent drug pair in dormancy (GRinf < -0.012)
min (most potent) GRinf in dormancy
min (most potent) GRinf in butyrate
E
butyrate + standard + valerate
rational +C2 design rules
1. potent drug pair in butyrate (GRinf < -0.38)
+
2. potent overall in standard (GRinf < -0.066)
mean (overall potency) of GRinf in standard
min (most potent) GRinf in butyrate
F
butyrate + standard + valerate
rational +C2 design rules
1. potent drug pair in butyrate (GRinf < -0.38)
+
2. synergistic drug pair in valerate (log2FIC50 < -0.23)
min (most synergistic) log2FIC50 in valerate
min (most potent) GRinf in butyrate
G
acidic + dormancy + cholesterol-high
rational +C2 design rules
1. synergistic drug pair in dormancy (log2FIC50 < -0.84)
+
2. additive/antagonistic overall in acidic (log2FIC50 > -0.062)
mean (overall synergy) log2FIC50 in acidic
min (most synergistic) log2FIC50 in dormancy
H
acidic + dormancy + cholesterol-high
rational +C2 design rules
1. synergistic drug pair in dormancy (log2FIC50 < -0.84)
+
2. additive/synergistic drug pair in cholesterol-high (log2FIC50 < 0.13)
min (most synergistic) log2FIC50 in cholesterol-high
min (most synergistic) log2FIC50 in dormancy

Figure S4. Individual feature univariate and decision tree analyses, related to Figure 3 and 4. (A-C) Univariate analysis of features using combined training and test data. Univariate feature analysis in SOC and BPaL models. (A) Box plots showing the distribution of values for drug interaction (log_2FIC_{50} and log_2FIC_{90}), and drug potency (E_{inf}, GR_{inf}, and AUC_{25} based on BPaL (green = +C2 and yellow = -C2) outcome. (B) Scatter plot of p-values for the Wilcoxon Rank Sum test evaluated for predicting SOC (-C1 vs +C1) and BPaL (-C2 vs +C2) outcomes. Features are colored by *in vitro* condition and shaped by metric type (circle, AUC_{25}; square, E_{inf}; downward triangle, GR_{inf}). P-values are corrected for multiple hypothesis testing within each outcome group (e.g., corrected for SOC comparison separate from BPaL comparison). Dashed lines show p=0.05. Features with FDR p-values <0.05 are annotated with extra information such as time (C or T for constant or terminal, respectively) and the summary statistic type (min, mean, or max). Linear regression line (solid black), confidence interval (shaded region), Pearson correlation coefficient (R) and associated p-value are indicated on plot. (C) Scatter plot of p-values from the Wilcoxon rank-sum tests contrasting values of individual drug interaction features across SOC (-C1 vs. +C1) and BPaL (-C2 vs. +C2) outcomes. Plot elements are analogous to those in panel B. Features are shaped by metric type (upward triangle, log_2FIC_{90}; diamond, log_2FIC_{50}). (D-H) Alternative ruleset scatter plots. Scatter plots of two metrics from each subset of conditions identified to be important for outperforming BPaL for each subset of conditions: (D) butyrate+dormancy+choleterol-high, (E and F) butyrate+standard+valerate, (G and H) acidic+dormancy+cholesterol-high. Plots are labeled as in Figure 4. Combinations are separated into those that were used in decision tree model training (circle, top-left), testing (triangle, top-right), or are candidates (square, bottom-left). Selected drug combinations are indicated with labels. Plot regions are colored based on the decision tree classification using thresholds (dashed lines) learned during training. Selected drug pair metric values are indicated along plot margins. Logic formatted rules are written in the bottom-right of each panel.

Figure S5

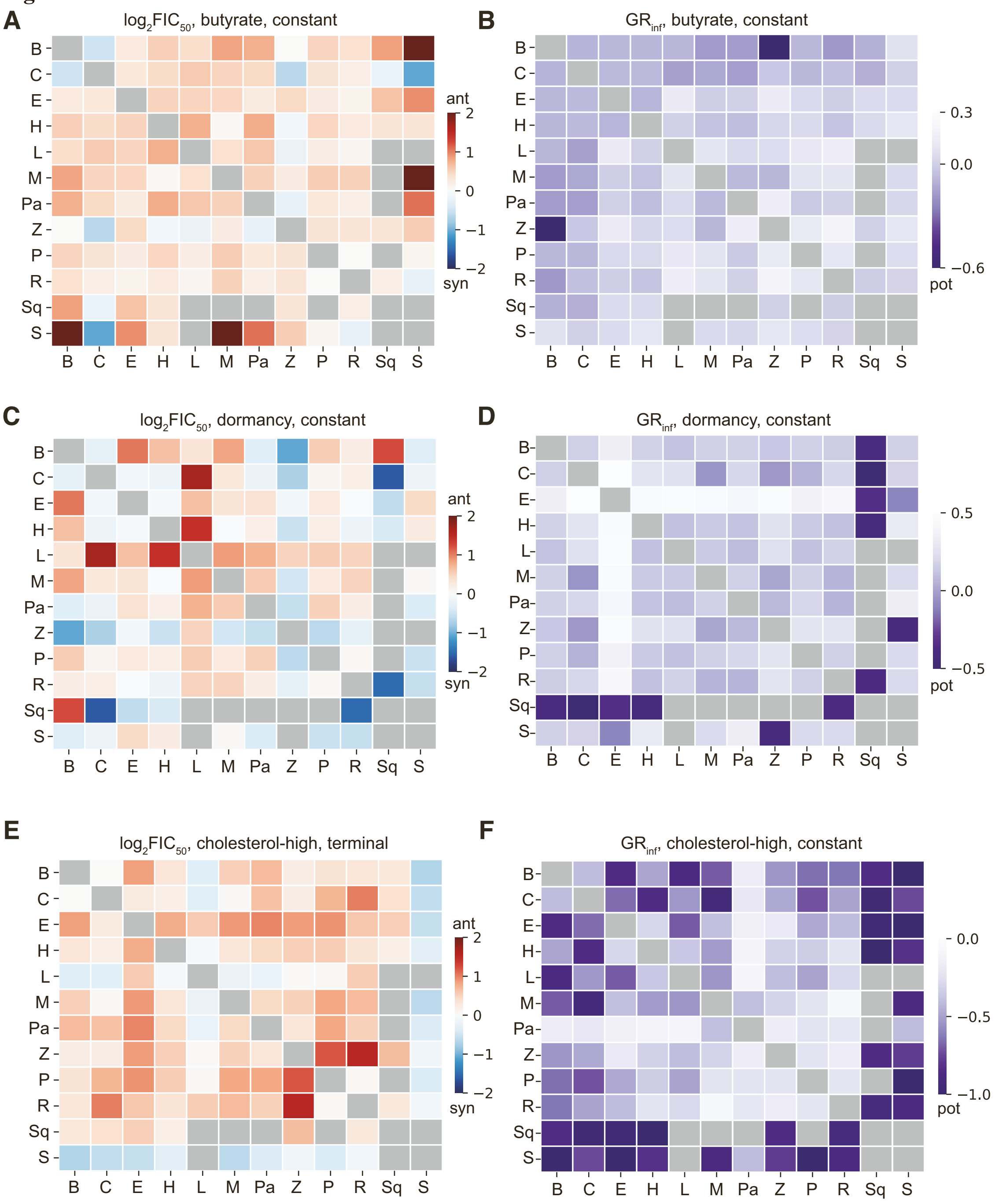

Figure S5. "At-a-glance" drug pair *in vitro* metric heatmaps, related to Figure 4. Heatmap of drug pair data for selected drug interaction (A, C, E) and potency (B, D, F) features for the conditions butyrate (A, B), dormancy (C, D), and cholesterol-high (E, F). Drugs are indicated along plot margins using abbreviations as in Table 1. Drug pair data are colored by their values for the indicated metric and condition.

Figure S6

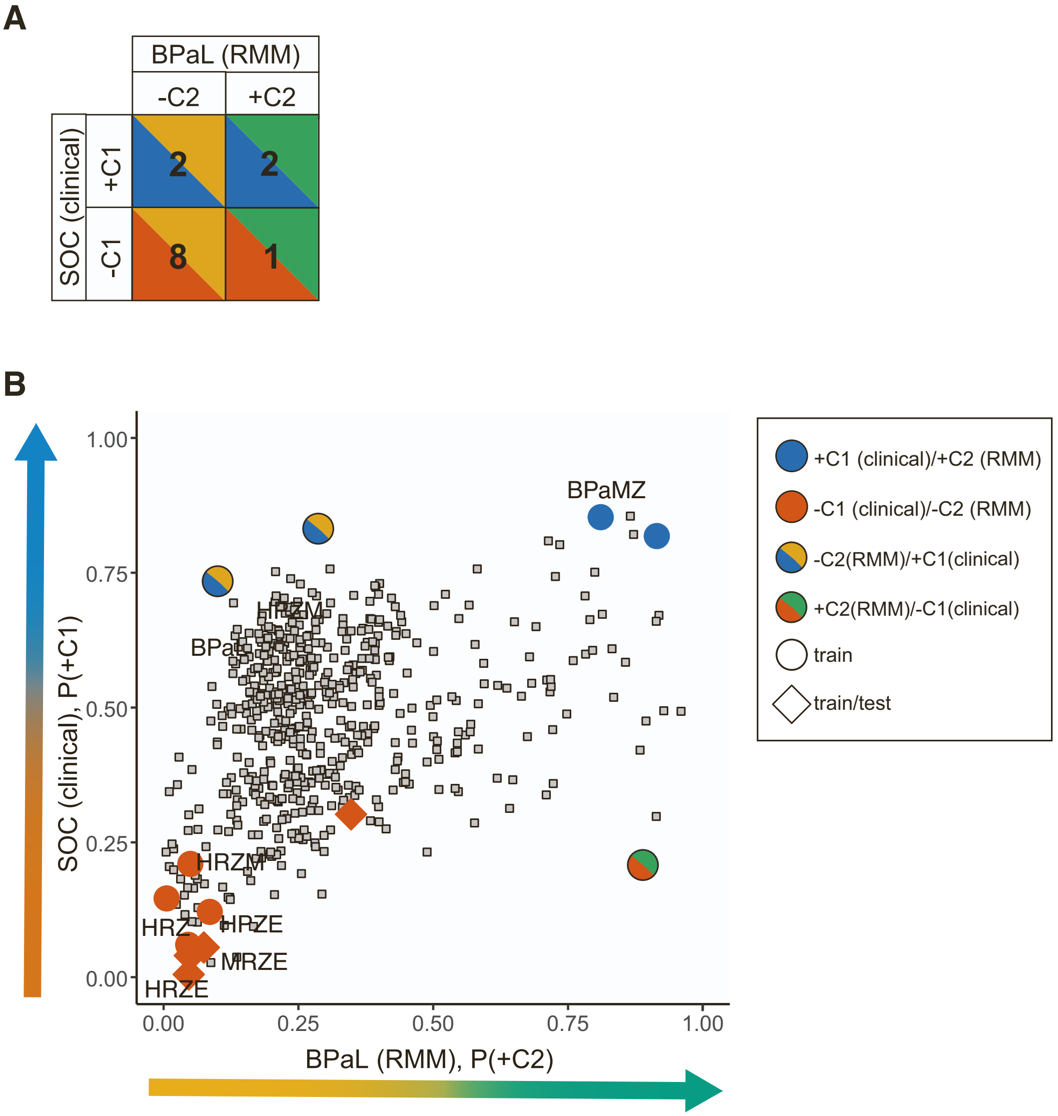

Figure S6. RMM predictions help stratify clinical SOC predictions, related to Figure 5. (A) Overlap in drug combination categorization between BPaL (RMM) and SOC (clinical) outcomes. Green/Blue and Orange/Red squares indicate treatment improvement agreement (+C2/+C1) between outcomes. Yellow/Blue and Green/Red squares highlight treatment improvement differences between outcome annotation (+C2/-C1 or -C2/+C1). (B) Probability scatter plot for BPaL model predictions (+C2 probability) and clinical model predictions (+C1 probability) using the butyrate+dormancy+cholesterol-high condition data. Annotated combinations are colored by clinical outcome when treatment improvement agrees, or split color is shown as in panel A. Model training combinations for both BPaL and clinical are labeled with circles. Combinations used for testing the BPaL model and training the clinical model training are labeled with diamonds. Candidate combinations (without annotations) are labeled with grey squares, and the number and percent of candidates in quadrants are indicated.

Figure S7

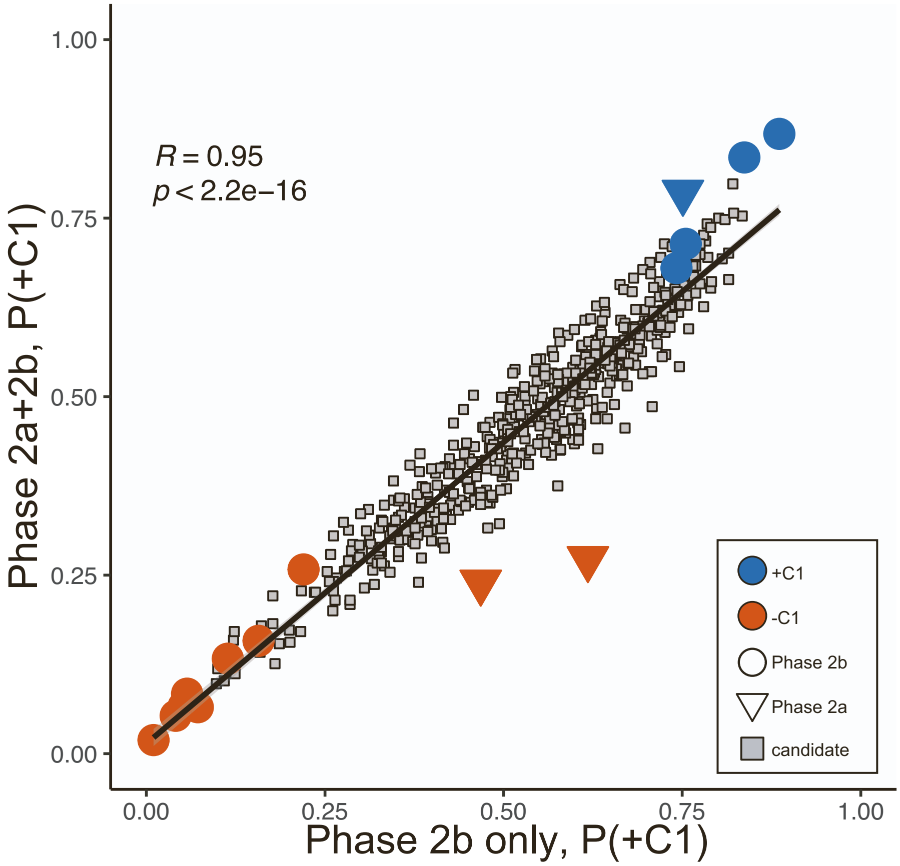

Figure S7. Prediction correlation from clinical models with and without Phase 2a trial combinations, related to Figure 5. Scatter plot of prediction probabilities from model trained with only Phase 2b trial combinations (12 combinations) and model trained with Phase 2a and Phase 2b trial combinations (15 combinations). Annotated combinations used for model training are indicated with circles (Phase 2b) and triangles (Phase 2a). Candidate combinations are in grey boxes. Linear regression line, Pearson correlation coefficient (R), and associated p-value are shown.

References

S1. Lee, B.Y., et al., *Ultra-rapid near universal TB drug regimen identified via parabolic response surface platform cures mice of both conventional and high susceptibility.* PLoS One, 2018. **13**(11): p. e0207469.

S2. Lee, B.Y., et al., *Drug regimens identified and optimized by output-driven platform markedly reduce tuberculosis treatment time.* Nat Commun, 2017. **8**: p. 14183.

S3. Williams, K., et al., *Sterilizing activities of novel combinations lacking first- and second-line drugs in a murine model of tuberculosis.* Antimicrob Agents Chemother, 2012. **56**(6): p. 3114-20.

S4. Diacon, A.H., et al., *Bactericidal activity of pyrazinamide and clofazimine alone and in combinations with pretomanid and bedaquiline.* Am J Respir Crit Care Med, 2015. **191**(8): p. 943-53.

S5. Tasneen, R., et al., *Contribution of the nitroimidazoles PA-824 and TBA-354 to the activity of novel regimens in murine models of tuberculosis.* Antimicrob Agents Chemother, 2015. **59**(1): p. 129-35.

S6. Tasneen, R., et al., *Sterilizing activity of novel TMC207- and PA-824-containing regimens in a murine model of tuberculosis.* Antimicrob Agents Chemother, 2011. **55**(12): p. 5485-92.

S7. Clemens, D.L., et al., *Artificial intelligence enabled parabolic response surface platform identifies ultra-rapid near-universal TB drug treatment regimens comprising approved drugs.* PLoS One, 2019. **14**(5): p. e0215607.

S8. Williams, K.N., et al., *Addition of PNU-100480 to first-line drugs shortens the time needed to cure murine tuberculosis.* Am J Respir Crit Care Med, 2009. **180**(4): p. 371-6.

S9. Tasneen, R., et al., *Contribution of Oxazolidinones to the Efficacy of Novel Regimens Containing Bedaquiline and Pretomanid in a Mouse Model of Tuberculosis.* Antimicrob Agents Chemother, 2016. **60**(1): p. 270-7.

S10. Xu, J., et al., *Contribution of Pretomanid to Novel Regimens Containing Bedaquiline with either Linezolid or Moxifloxacin and Pyrazinamide in Murine Models of Tuberculosis.* Antimicrob Agents Chemother, 2019. **63**(5).

S11. Berg, A., et al., *Model-Based Meta-Analysis of Relapsing Mouse Model Studies from the Critical Path to Tuberculosis Drug Regimens Initiative Database.* Antimicrob Agents Chemother, 2022. **66**(3): p. e0179321.

S12. Mudde, S.E., et al., *Predictive Modeling to Study the Treatment-Shortening Potential of Novel Tuberculosis Drug Regimens, Toward Bundling of Preclinical Data.* The Journal of Infectious Diseases, 2021.

S13. Li, S.Y., et al., *Bactericidal and Sterilizing Activity of a Novel Regimen with Bedaquiline, Pretomanid, Moxifloxacin, and Pyrazinamide in a Murine Model of Tuberculosis.* Antimicrob Agents Chemother, 2017. **61**(9).

S14. Tweed, C.D., et al., *Bedaquiline, moxifloxacin, pretomanid, and pyrazinamide during the first 8 weeks of treatment of patients with drug-susceptible or drug-resistant pulmonary tuberculosis: a multicentre, open-label, partially randomised, phase 2b trial.* Lancet Respir Med, 2019. **7**(12): p. 1048-1058.

S15. Andries, K., T. Gevers, and N. Lounis, *Bactericidal potencies of new regimens are not predictive of their sterilizing potencies in a murine model of tuberculosis.* Antimicrob Agents Chemother, 2010. **54**(11): p. 4540-4.

S16. Saini, V., et al., *Treatment-Shortening Effect of a Novel Regimen Combining Clofazimine and High-Dose Rifapentine in Pathologically Distinct Mouse Models of Tuberculosis.* Antimicrob Agents Chemother, 2019. **63**(6).
S17. Ammerman, N.C., et al., *Impact of Clofazimine Dosing on Treatment Shortening of the First-Line Regimen in a Mouse Model of Tuberculosis.* Antimicrob Agents Chemother, 2018. **62**(7).
S18. Tyagi, S., et al., *Clofazimine shortens the duration of the first-line treatment regimen for experimental chemotherapy of tuberculosis.* Proc Natl Acad Sci U S A, 2015. **112**(3): p. 869-74.
S19. Mourik, B.C., et al., *Improving treatment outcome assessment in a mouse tuberculosis model.* Sci Rep, 2018. **8**(1): p. 5714.
S20. Rosenthal, I.M., et al., *Dose-ranging comparison of rifampin and rifapentine in two pathologically distinct murine models of tuberculosis.* Antimicrob Agents Chemother, 2012. **56**(8): p. 4331-40.
S21. Bonnett, L.J., et al., *Comparing the Efficacy of Drug Regimens for Pulmonary Tuberculosis: Meta-analysis of Endpoints in Early-Phase Clinical Trials.* Clin Infect Dis, 2017. **65**(1): p. 46-54.
S22. Dorman, S.E., et al., *Substitution of rifapentine for rifampin during intensive phase treatment of pulmonary tuberculosis: study 29 of the tuberculosis trials consortium.* J Infect Dis, 2012. **206**(7): p. 1030-40.
S23. Dorman, S.E., et al., *Four-Month Rifapentine Regimens with or without Moxifloxacin for Tuberculosis.* N Engl J Med, 2021. **384**(18): p. 1705-1718.
S24. Li, S.Y., et al., *Evaluation of moxifloxacin-containing regimens in pathologically distinct murine tuberculosis models.* Antimicrob Agents Chemother, 2015. **59**(7): p. 4026-30.
S25. Mourik, B.C., et al., *Assessment of Bactericidal Drug Activity and Treatment Outcome in a Mouse Tuberculosis Model Using a Clinical Beijing Strain.* Antimicrob Agents Chemother, 2017. **61**(10).
S26. Gillespie, S.H., et al., *Four-month moxifloxacin-based regimens for drug-sensitive tuberculosis.* N Engl J Med, 2014. **371**(17): p. 1577-87.
S27. Boeree, M.J., et al., *High-dose rifampicin, moxifloxacin, and SQ109 for treating tuberculosis: a multi-arm, multi-stage randomised controlled trial.* Lancet Infect Dis, 2017. **17**(1): p. 39-49.
S28. Dorman, S.E., et al., *Substitution of moxifloxacin for isoniazid during intensive phase treatment of pulmonary tuberculosis.* Am J Respir Crit Care Med, 2009. **180**(3): p. 273-80.
S29. Tweed, C.D., et al., *A partially randomised trial of pretomanid, moxifloxacin and pyrazinamide for pulmonary TB.* Int J Tuberc Lung Dis, 2021. **25**(4): p. 305-314.
S30. Rustomjee, R., et al., *A Phase II study of the sterilising activities of ofloxacin, gatifloxacin and moxifloxacin in pulmonary tuberculosis.* Int J Tuberc Lung Dis, 2008. **12**(2): p. 128-38.
S31. Conde, M.B., et al., *Moxifloxacin versus ethambutol in the initial treatment of tuberculosis: a double-blind, randomised, controlled phase II trial.* Lancet, 2009. **373**(9670): p. 1183-9.
S32. Conde, M.B., et al., *A Phase 2 Randomized Trial of a Rifapentine plus Moxifloxacin-Based Regimen for Treatment of Pulmonary Tuberculosis.* PLoS One, 2016. **11**(5): p. e0154778.

S33. Lee, J.K., et al., *Substitution of ethambutol with linezolid during the intensive phase of treatment of pulmonary tuberculosis: a prospective, multicentre, randomised, open-label, phase 2 trial.* Lancet Infect Dis, 2019. **19**(1): p. 46-55.

S34. Lanoix, J.P., F. Betoudji, and E. Nuermberger, *Sterilizing Activity of Pyrazinamide in Combination with First-Line Drugs in a C3HeB/FeJ Mouse Model of Tuberculosis.* Antimicrob Agents Chemother, 2016. **60**(2): p. 1091-6.

S35. Nuermberger, E.L., et al., *Moxifloxacin-containing regimen greatly reduces time to culture conversion in murine tuberculosis.* Am J Respir Crit Care Med, 2004. **169**(3): p. 421-6.

S36. Nuermberger, E., et al., *Combination chemotherapy with the nitroimidazopyran PA-824 and first-line drugs in a murine model of tuberculosis.* Antimicrob Agents Chemother, 2006. **50**(8): p. 2621-5.

S37. Rosenthal, I.M., et al., *Isoniazid or moxifloxacin in rifapentine-based regimens for experimental tuberculosis?* Am J Respir Crit Care Med, 2008. **178**(9): p. 989-93.

S38. Rosenthal, I.M., et al., *Daily dosing of rifapentine cures tuberculosis in three months or less in the murine model.* PLoS Med, 2007. **4**(12): p. e344.

S39. De Groote, M.A., et al., *Comparative studies evaluating mouse models used for efficacy testing of experimental drugs against Mycobacterium tuberculosis.* Antimicrob Agents Chemother, 2011. **55**(3): p. 1237-47.

S40. De Groote, M.A., et al., *Importance of confirming data on the in vivo efficacy of novel antibacterial drug regimens against various strains of Mycobacterium tuberculosis.* Antimicrob Agents Chemother, 2012. **56**(2): p. 731-8.

S41. Nuermberger, E., et al., *Powerful Bactericidal and Sterilizing Activity of a Regimen Containing PA-824, Moxifloxacin, and Pyrazinamide in a Murine Model of Tuberculosis.* Antimicrobial Agents and Chemotherapy, 2008. **52**(4): p. 1522-1524.

S42. Nuermberger, E.L., et al., *Moxifloxacin-containing regimens of reduced duration produce a stable cure in murine tuberculosis.* Am J Respir Crit Care Med, 2004. **170**(10): p. 1131-4.
