# Supplementary figures and images for: Design principles to assemble drug combinations for effective tuberculosis therapy using interpretable pairwise drug response measurements
Source: Cell Rep Med. 2022 Sep 8;3(9):100737. doi: 10.1016/j.xcrm.2022.100737 (PMC9512659; doi:10.1016/j.xcrm.2022.100737)

# ROC plot

bsv in vitro model

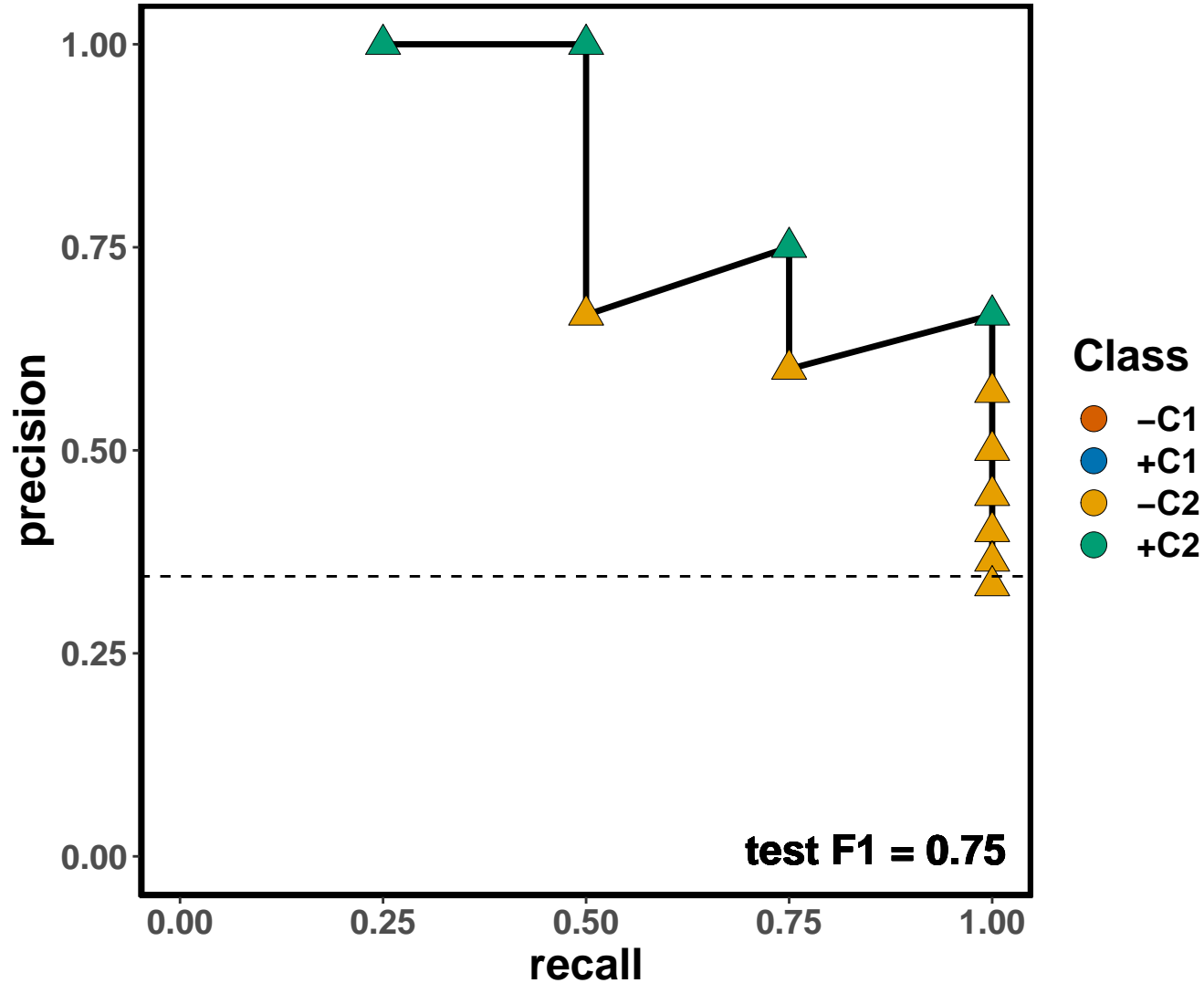

Supplement: Data S6. Original code for generating figures and tables, related to Figures 2-5, S2-S7, and Data S1, S3-S5 [file mmc7.zip › Data S6/Figure S3/FigureS3B_BPaL_PR.pdf]

# ROC plot

bsv in vitro model

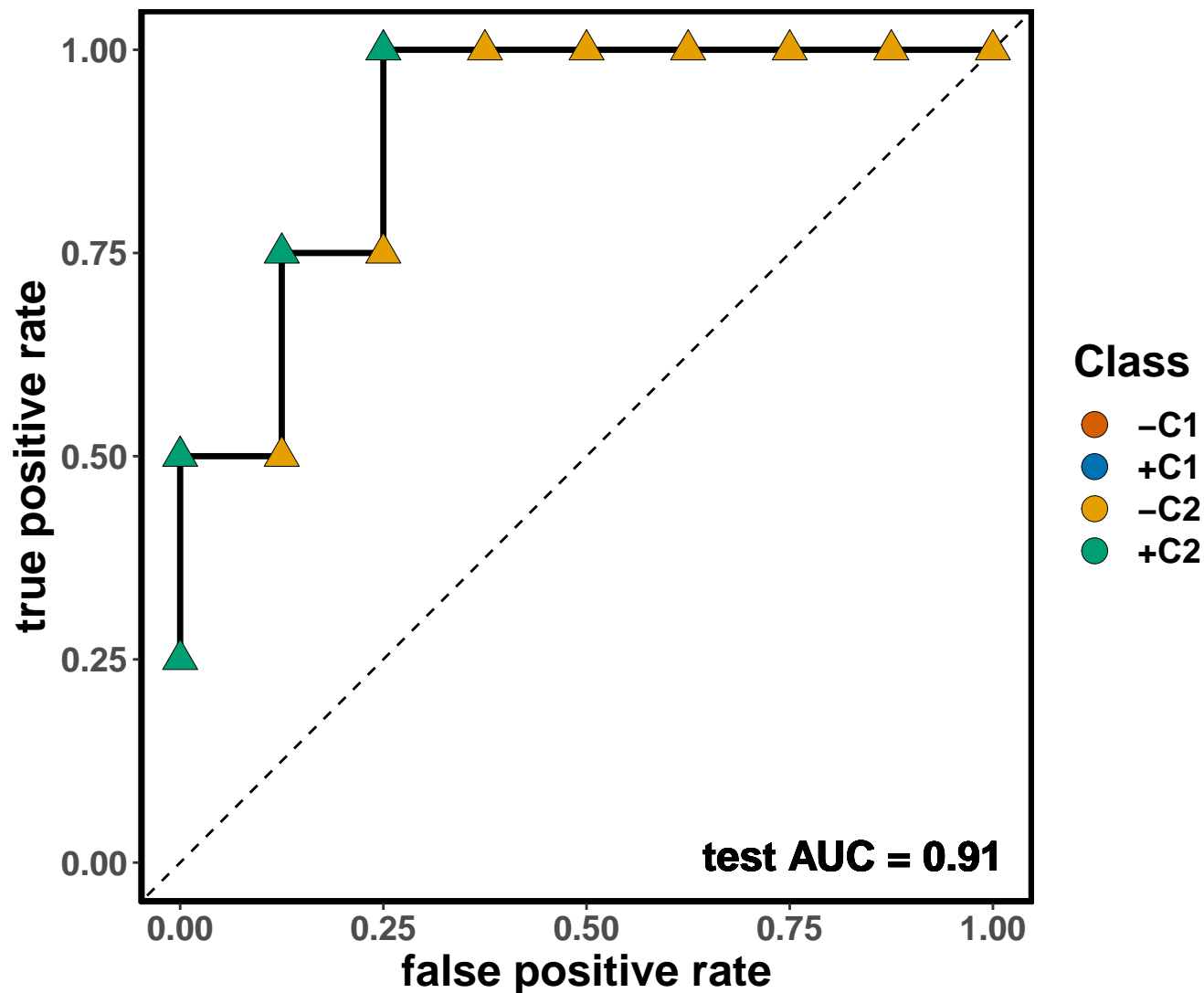

Supplement: Data S6. Original code for generating figures and tables, related to Figures 2-5, S2-S7, and Data S1, S3-S5 [file mmc7.zip › Data S6/Figure S3/FigureS3B_BPaL_ROC.pdf]
